# Genetic architecture and functional consequences of lateral root length in maize

**DOI:** 10.1093/jxb/erag130

**Published:** 2026-03-12

**Authors:** Fabio Guffanti, Daniela Scheuermann, Claude Urbany, Stefan Reuscher, Thomas Presterl, Milena Ouzunova, Silvio Salvi, Caroline Callot, Chris-Carolin Schön

**Affiliations:** Plant Breeding, TUM School of Life Sciences, Technical University of Munich, D-85354 Freising, Germany; KWS SAAT SE & Co. KGaA, D-37574 Einbeck, Germany; KWS SAAT SE & Co. KGaA, D-37574 Einbeck, Germany; KWS SAAT SE & Co. KGaA, D-37574 Einbeck, Germany; KWS SAAT SE & Co. KGaA, D-37574 Einbeck, Germany; KWS SAAT SE & Co. KGaA, D-37574 Einbeck, Germany; Department of Agricultural and Food Sciences, University of Bologna, 40127 Bologna, Italy; CNRGV, INRAE, French Plant Genomic Resource Center, Castanet Tolosan 31326, France; Plant Breeding, TUM School of Life Sciences, Technical University of Munich, D-85354 Freising, Germany; INRAE-Montpellier, France

**Keywords:** breeding, diversity, genes, genetic architecture, landraces, lateral roots, nitrogen, phosphorus, QTL, *Zea mays*

## Abstract

Understanding the genetic basis of root architecture and its relevance for crop productivity can contribute to the sustainable intensification of agriculture. Leveraging the phenotypic and allelic diversity of an Austrian maize landrace, we dissected the genetic basis of lateral root (LR) length across developmental stages. LR length, a relevant trait for breeding resource-efficient varieties, showed high heritability in our experiments. We discovered eight quantitative trait loci (QTLs) for LR length at the reproductive stage R2, overlapping with four QTLs at stage R6 but not with QTLs detected at vegetative stage V6, suggesting that the genetic regulation of LR length might differ in vegetative and reproductive stages. We fine-mapped *qlr1*, the most significant QTL for LR length, to a region of 2.3 Mb containing 46 annotated genes. Based on whole-genome sequence and comparative genomics analyses, we suggest a candidate gene underlying *qlr1*. Additionally, we examined the impact of nitrogen, phosphorus, and irrigation treatments on root and shoot development, finding that LR length positively correlates with biomass accumulation under optimal nutrient supply but not under nitrogen stress. Our work provides insights into the genetic regulation of LR length in maize and its relevance for the adaptation to diverse growing environments.

## Teaser

Multiple QTLs affect lateral root length in maize at different developmental stages. Genotypes with longer lateral roots exhibited similar or higher shoot dry weight across diverse field conditions.

## Introduction

Roots are critical to crop productivity and sustainability, as water and nutrient availability impair yield potential of all major crops, including maize (*Zea mays* L.) (Mueller *et al.*, 2012; Lynch, 2022). Breeding for root traits that enhance resource acquisition, particularly in low-fertility soils, is suggested to play an important role in the sustainable intensification of agriculture (Lynch, 2007). The maize root system comprises embryonic roots, namely the primary and seminal roots, and post-embryonic roots originating from the shoot (Hochholdinger *et al.*, 2018). Shoot-borne roots (also called nodal roots) develop in consecutive shoot whorls (or nodes) and dominate the adult maize root system. They are classified as crown and brace roots depending on their development below or above the soil surface (Hochholdinger *et al.*, 2004).

Lateral roots (LRs), which emerge post-embryonically from all root types, constitute most of the total root length and exhibit high responsiveness to environmental stimuli, such as water, nutrients, and microbial interactions (Yu *et al.*, 2014, 2016; Viana *et al.*, 2022). In young maize plants, shoot-borne roots and their LRs already constitute most of the total root length and take up the majority of the water (Singh *et al.*, 2010; Ahmed *et al.*, 2018). They reach their maximum cumulative length between flowering time and 2 weeks after flowering, corresponding to developmental stage R2 in field-grown maize (Peng *et al.*, 2010, 2012), providing anchorage and facilitating soil resource acquisition throughout the life cycle of the plant (Hochholdinger *et al.*, 2004).

LR length, branching frequency, and plasticity are important breeding targets to improve productivity, especially under suboptimal growing conditions. Maize recombinant inbred lines (RILs) differing in LR length and branching frequency at flowering have been shown to exhibit differential biomass accumulation and yield in the field under irrigation, nitrogen, and phosphorus treatments (Zhan *et al.*, 2015; Zhan and Lynch, 2015; Jia *et al.*, 2018). Genotypes with few and long LRs performed better under low water and nitrogen availability, while those with short and dense LRs performed better in low phosphorus conditions.

In maize, genes affecting LR initiation, elongation, and number have been described for primary and seminal roots (Woll *et al.*, 2005; von Behrens *et al.*, 2011; Lu *et al.*, 2019; Gautam *et al.*, 2021; Baer *et al.*, 2023; Zhang *et al.*, 2023). In addition, the number of quantitative trait locus (QTL) mapping studies for shoot-borne root traits has increased in recent years, identifying QTLs of small to moderate effect, and suggesting a quantitative genetic architecture. These studies have primarily focused on axial roots (Z.H. Zhang *et al.*, 2018; Wang *et al.*, 2021; Li *et al.*, 2024), while LR traits such as length and density have been explored less (Schneider *et al.*, 2020).

A valuable resource to study maize root traits is the genetic diversity offered by landraces, which might harbour new, favourable alleles compared with elite material (Mayer *et al.*, 2020). A library of doubled-haploid (DH) lines derived from three European maize landraces showed striking genetic diversity for many agronomic traits relevant for breeding (Mayer *et al.*, 2017; Hoelker *et al.*, 2019). This natural mutant collection was successfully used to identify a QTL related to early plant development, including the discovery of the underlying causal gene and elucidation of its functional consequences and allelic diversity (Urzinger *et al.*, 2025).

In the present work, we investigated the natural variation of shoot-borne root architecture in DH lines derived from the Austrian maize landrace ‘Kemater Landmais Gelb’ (KE). We developed a series of extensively characterized bi-parental populations derived from two DH lines with large differences in their LR length and similar genomic background. The objectives of this study were to uncover the genetic architecture of LR length in shoot-borne roots during vegetative and reproductive stages, fine-map and characterize the most significant QTL for LR length, and evaluate the impact of LR length on shoot biomass accumulation under different phosphorus, nitrogen, and water regimes.

## Materials and methods

### Plant material

In previous work, a library of >1000 DH lines was generated, sampling a large number of individuals from three European maize landraces (Mayer *et al.*, 2022). They were chosen to represent the molecular diversity of a comprehensive panel of 35 flint landraces, covering a broad geographical region of Europe (Mayer *et al.*, 2017). A detailed description of the development and molecular and phenotypic characterization of the landrace-derived DH library is given in Hoelker *et al.* (2019). Briefly, DH lines were genotyped with the 600k Affymetrix® Axiom® Maize Genotyping Array (Unterseer *et al.*, 2014), hereinafter referred to as the 600k array, and phenotyped for agronomic traits in up to 11 field environments. This resource served as the basis for the eight experiments (E0–E7) presented in this study. In E0, the aim was to screen the adult root architecture of DH lines derived from the Austrian landrace KE to select parental lines for generating a bi-parental population. We analysed the mature root system (developmental stage R2) of 18 DH KE lines forming nine pairs, each sharing a similar genomic background but carrying contrasting haplotypes associated with lodging and/or seedling root traits in earlier genome-wide association studies (GWAS) (Mayer *et al.*, 2020).

**Fig. 1.**
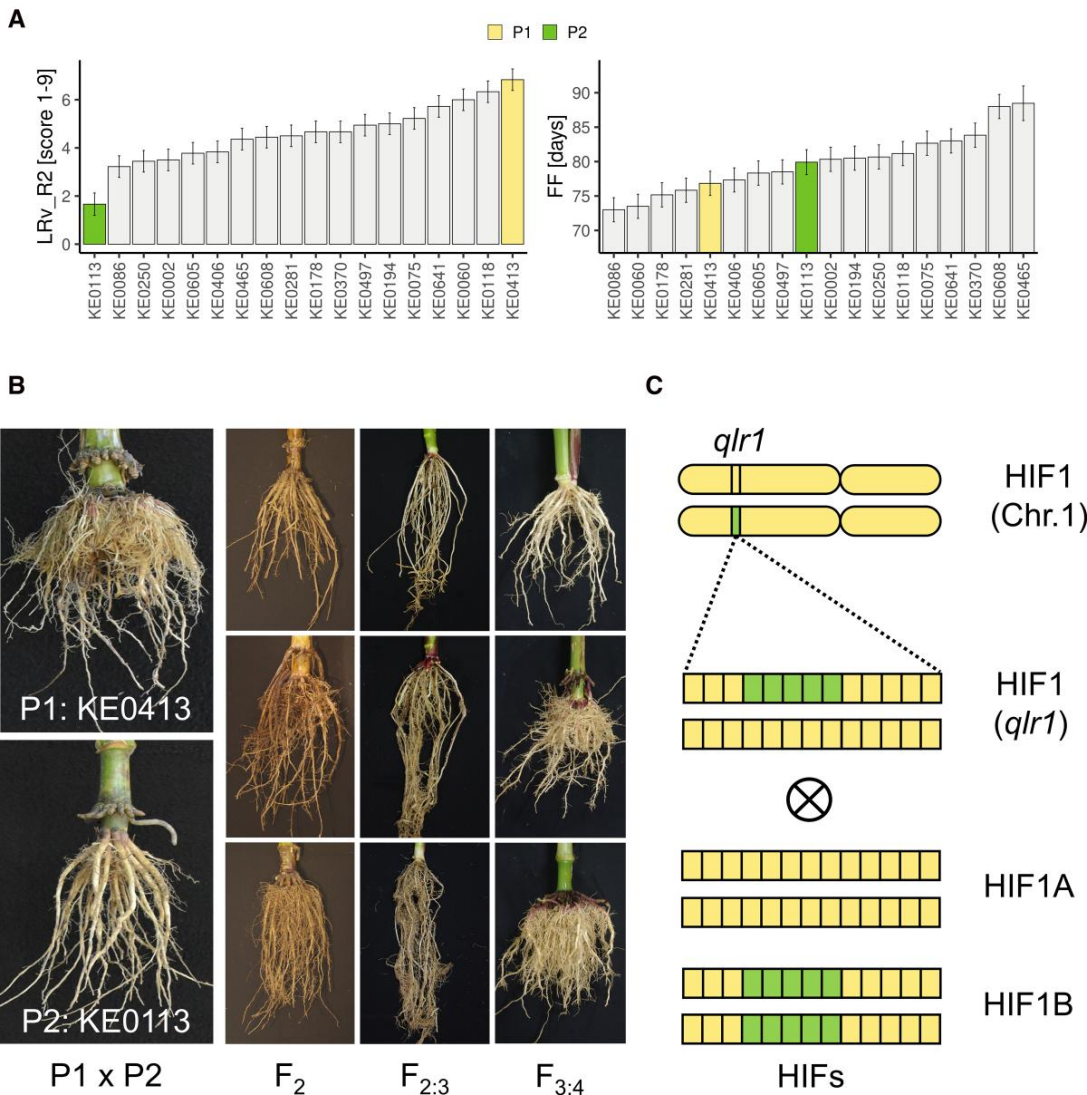
Selection of parental DH lines and development of plant material. (A) Visually scored LR length at stage R2 (LRv_R2) and flowering time (FF) of 18 landrace-derived DH lines evaluated in experiment E0. Bars represent adjusted means across two locations ±SEs. (B) P1 (KE0413) and P2 (KE0113) were crossed to develop QTL mapping populations segregating for LRv evaluated in E1–E3. LRv was evaluated on F_2_ individuals in E1 at developmental stage R6, on F_2:3_ progenies in E2 at stage V6, and on F_3:4_ progenies in E3 at stage R2. (C) Example of the development of one HIF (HIF1) used for fine-mapping *qlr1.* HIFs were derived by self-pollination of F_3_, F_4_, or F_5_ individuals with heterozygous fragments of *qlr1* (in this case from marker 4 to marker 9). Each resulting HIF is represented by two contrasting lines (HIFA and HIFB) homozygous for the P1 or the P2 allele while sharing a similar genomic background. Vertical segments indicate schematically the position of the 14 KASP markers used to genotype the HIFs. A total of 43 HIFs, differing with respect to the size and position of contrasting fragments, was evaluated in the fine-mapping experiments E4–E7.

One pair of these DH lines, KE0413 (P1) and KE0113 (P2), with similar genomic background [74 978 polymorphic single nucleotide polymorphisms (SNPs) out of 501 124 SNPs], similar flowering time, and contrasting LR architecture, was crossed to generate the populations used in this study, comprising F_2_ individuals, RILs, and heterogeneous inbred families (HIFs) (Fig. 1). In E1–E7, we evaluated the populations derived from the bi-parental cross. In E1–E3 the aim was to map QTLs for root and shoot traits at different developmental stages in F_2_ individuals, and F_2:3_ and F_3:4_ RILs. In E4–E7, the aim was to fine-map and characterize *qlr1*, the most significant of the QTLs for LR length identified in E1–E3, in segregating HIFs.

### Development of recombinant inbred lines and heterogeneous inbred families

The F_2_ segregating population comprised 554 individuals. Through self-pollination, 150 F_2:3_ and 285 F_3:4_ RILs were derived from F_2_ and F_3_ individuals, respectively. Genotyping was performed on F_2_ and F_3_ individuals using a custom 15 K SNP Illumina array proprietary to KWS SAAT SE & Co. KGaA (hereinafter referred to as the 15 K SNP array).

**Fig. 2.**
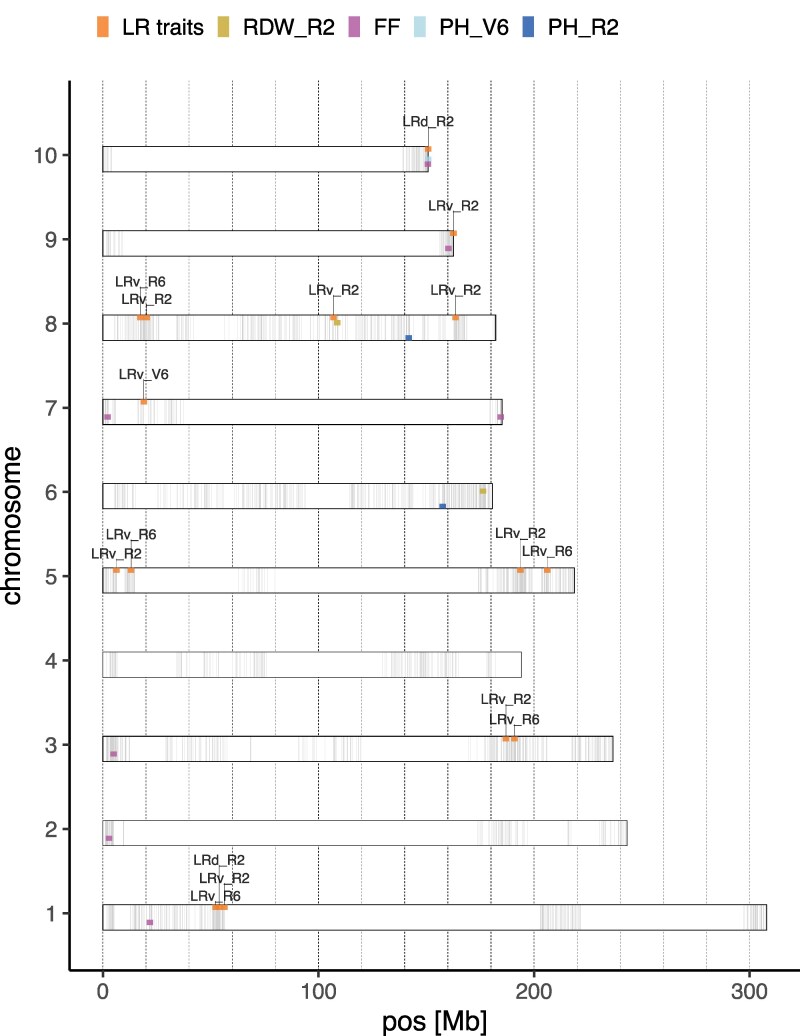
QTLs for root and agronomic traits. QTL positions on the B73v5 physical map for visually scored lateral root (LR) length at developmental stages V6, R2, and R6 (LRv_V6, LRv_R2, and LRv_R6), average LR length assessed with DIRT at stage R2 (LRd_R2), root dry weight at stage R2 (RDW_R2), flowering time (FF), and plant height at stages V6 and R2 (PH_V6, PH_R2). Rectangles represent the 10 chromosomes; grey segments indicate polymorphic markers between P1 and P2 used for genetic map construction and QTL mapping. Coloured points denote QTLs associated with different traits. QTLs for LR-related traits are labelled.

Based on the results of the QTL mapping experiments E1–E3 (Fig. 2), the most significant QTL for LR length on Chr. 1 (*qlr1*) was targeted for fine-mapping. A total of 14 KASP (Kompetitive allele-specific PCR) markers polymorphic between the parental lines and spanning a 26 Mb interval on Chr. 1 (from AX-91333740, pos. 30 671 047 on the B73v5 physical map, to AX-90529307, pos. 56 348 234) were synthesized based on the 600 K array probe sequences to identify recombination breakpoints within *qlr1*. Individual F_3_, F_4_, or F_5_ plants carrying different heterozygous fragments of *qlr1* at the genotyped KASP markers were self-pollinated to generate 43 HIFs (Fig. 1C; Supplementary Table S1). The resulting progenies, segregating for the marker alleles of the respective fragments in a near-isogenic background, were examined in the fine-mapping experiments E4–E7, either directly or following seed multiplication. Additionally, two F_4_ individuals homozygous for the P1 allele at all 14 KASP markers within *qlr1* and two F_4_ individuals homozygous for the P2 allele for 13 KASP markers within *qlr1* (from 34 Mb to 56 Mb) were self-pollinated twice to generate F_4:6_ RILs contrasting for the entire region of *qlr1* to be included in E4 and E5.

### Growth conditions and experimental design

Details for all experiments with respect to plant material, treatments, and replications are summarized in Table 1. Further details concerning soil properties and elemental composition, field coordinates, and experimental design are provided in Supplementary Table S2. This study targets temperate maize-growing environments typical of Central Europe, reflecting field conditions with contrasting nitrogen (N), phosphorus (P), and water availability. Accordingly, the field sites in Germany fell within the typical growing range of early- to medium-maturity maize. Complementary greenhouse experiments were conducted in winter.

**Table 1.** Detailed information on experiments E0–E7

E	Y	L	T	PT	no G	r G	no P	Traits	*h* ^2^ (LRv)
E0	2020	FS	CTR	DH	18	3	180	LRv, FF, PH	
		BOL	CTR		18	3	180		
Across*^a^*					18	6	360		0.87
E1	2021	EIN	CTR	F_2_	554	1	569	LRv	
E2	2022	GH	CTR	F_2:3_ RILs	150	5	800	LRv	0.43
E3	2022	VIH	CTR	F_3:4_ RILs	285	1	900	LRv, LRd, LRBFd, RDW, FF, PH, SDW	
		VIH	LP		285	1	900		
		EIN	CTR		285	1	900		
Across*^a^*					285	3	2700		0.74
E4	2023	ROG	CTR	*qlr1* HIFs, F_4:6_ RILs	55	4	720	LRv, LRd, LRBFd, RDW, FF, PH, SDW	
		ROG	LN		55	4	720		
Across*^a^*					55	8	1440		0.83
E5	2023	FS	CTR	*qlr1* HIFs, F_4:6_ RILs	8	3	90	LRv, LRd, LRBFd, RDW, FF, PH, SDW	
		FS	WS		8	3	90		
Across*^a^*					8	6	180		0.89
E6	2023	GH	CTR	*qlr1* HIFs	30	6.9*^b^*	240	LRv, LRd, LRBFd, RDW, FF, PH, SDW	0.45
E7	2024	GH	CTR	*qlr1* HIFs	18	12	240	LRv, LRd, LRBFd, RDW, FF, PH, SDW	0.86
E4–E7				*qlr1* HIFs, F_4:6_ RILs	95*^c^*	9.6*^b^*	2100	LRv, LRd, LRBFd, RDW, FF, PH, SDW	0.75

Given are the year (Y) and location (L) of evaluation, applied treatment (T), progeny type (PT), number of genotypes (no G), number of replications of the genotypes (r G), total number of plants measured including genotypes and checks (no P), traits included for QTL mapping, and entry mean heritability of visually scored LR length [*h*^2^ (LRv)]

Location codes: FS, Freising (Germany); BOL, Bologna (Italy); EIN, Einbeck (Germany); GH, greenhouse; VIH, Viehhausen (Germany); ROG, Roggenstein (Germany).

Treatments codes: CTR, control; LP, low phosphorus; LN, low nitrogen; WS, water stress.

Trait codes: LRv, visual scoring of LR length; LRd, DIRT assessment of LR length; LRBFd, DIRT assessment of LR branching frequency; RDW, root dry weight; FF, flowering time; PH, plant height; SDW, shoot dry weight.

^*a*^Across trials, for experiment comprising more trials.

^*b*^Average number of replications.

^*c*^Total of 95 genotypes evaluated across four experiments E4–E7.

In E0, the 18 DH KE lines and two flint inbred lines were evaluated in field trials in two locations (Bologna, Italy and Freising, Germany) in 2020 for root traits at stage R2 and agronomic traits throughout development. In all subsequent experiments, P1 and P2 were included as check genotypes. In E1, E3, and E4, three additional flint check inbred lines were included.

In E1, 554 F_2_ individual plants were evaluated in one field location (Einbeck, Germany) in 2021 for root traits at stage R6. In E2, 150 F_2:3_ progenies were evaluated in the greenhouse in 2022 for root and shoot traits at the vegetative stage V6. In E3, 285 F_3:4_ RILs were evaluated in three field trials in two locations (Viehhausen and Einbeck, Germany) in 2022 for root traits at stage R2 and agronomic traits throughout development. In Viehhausen, two P treatments were applied in the same field: in the control trial 100 kg P ha^–1^ were supplied; in the low P trial, no P was supplied. The content of P in the untreated trial was determined to be 0.8 mg per 100 g before sowing with the CAL method (Schüller, 1969).

In E4, a total of 55 progenies from the bi-parental cross comprising 25 HIFs (12 F_3:5_ and 13 F_4:6_) and 4 F_4:6_ RILs (contrasting for *qlr1*, two RILs with P1 alleles and two RILs with P2 alleles) were evaluated under two N treatments in separate trials in the same field (Roggenstein, Germany) in 2023 for root traits at stage R2 and agronomic traits throughout development. In the control trial, 170 kg N ha^–1^ were supplied; in the low N trial, no N was supplied. The content of mineral N in the untreated field was determined to be 33 kg ha^–1^ before sowing with the N_min_ method (Wehrmann and Scharpf, 1986).

In E5, a subset of eight progenies from the bi-parental cross tested in E4, consisting of three F_4:6_ HIFs and two F_4:6_ RILs (contrasting for *qlr1*, one RIL with P1 alleles and one RIL with P2 alleles) were evaluated under two irrigation treatments in two separate trials in the same location (Freising, Germany) for root traits at stage R2 and agronomic traits throughout development. The water stress (WS) trial was performed in a rainout shelter, where water was exclusively supplied by irrigation. Plants in both trials were grown under well-watered conditions until 52 d after sowing, corresponding to 16–26 d before tasselling depending on the genotype. Thereafter, irrigation was restricted in the WS trial, with a total of 94.5 l m^−2^ applied in 15 irrigation events until maturity, each supplying 4.5–9 l m^−2^. The control trial was rain-fed and supplemented with irrigation as needed to fully meet crop evapotranspiration, totalling 331 l m^−2^.

In E6, a total of 13 F_3:4_ HIFs were evaluated in the greenhouse in 2023 for root traits at stage R2 and agronomic traits throughout development. In E7, nine HIFs (seven F_4:6_ and two F_5:7_) were evaluated in the greenhouse in 2024 for root traits at stage R2 and agronomic traits throughout development.

In the field experiments, plant protection, irrigation, herbicide, and fertilizer application were conducted according to standard agronomic practice for the respective soil types and locations unless specified otherwise. The experimental units were single- or double-row (only E4) plots of 20 plants per 3 m row, with a row width of 75 cm. In the greenhouse, the conditions were maintained at 25–35 °C/18–20 °C day/night, 40% relative humidity, and a light intensity of 600 μmol m^−2^ s^−1^ was supplied by ceramic metal-halide lamps in addition to natural light. Experimental units were single plants grown in pots of 3 litres (top diameter: 16 cm, height: 20 cm in E2) or of 15 litres (top diameter: 33 cm, height: 22 cm, in E6 and E7). The growing substrate consisted of a mixture of C-700 Stender (https://stender.de/) potting substrate (sieved with 10 mm mesh) and sand (0.7–1.2 mm) in a ratio of 75/25 (v/v).

### Phenotypic data collection

We measured root and shoot traits at different developmental stages to assess their responses and relationships under diverse growing conditions. For the assessment of root traits, the rootstocks were harvested and processed as described by Trachsel *et al.* (2011). In the field experiments (E0, E3, E4, and E5), three plants per plot were selected to be representative of the overall appearance of the plot, and a soil cylinder of ∼25 cm depth and 40 cm diameter was excavated for each rootstock. In the greenhouse experiments (E2, E6, and E7), rootstocks were extracted from the pots. In both settings, the stem was severed 10 cm above the soil surface, and the shoot was used to determine biomass-related traits. Soil and substrate adhering to the rootstocks were gently removed, after which the rootstocks were carefully washed to minimize root breakage. The cleaned rootstocks were then photographed together with one or two excised roots from the last developed whorl on a black background using a digital camera (Pentax K7, Pentax Ricoh Imaging Company, Ltd, Japan) in a dark room under controlled artificial lighting (Supplementary Fig. S1). The root pictures were analysed with the software DIRT (Digital Imaging of Root Traits) (Das *et al.*, 2015), which computed 34 root traits from the rootstock and 11 traits from the excised roots. In this study, we focused on two LR-related traits derived from the excised roots: average LR length (LRd, mm) and average LR branching frequency (LRBFd, branches cm^–1^), with ‘d’ indicating DIRT. In addition, LR length was visually (v) scored from the images of the rootstock (LRv, 1–9 score; the scoreboard used for LRv is shown in Supplementary Fig. S2). Rootstock fresh and dry weight (RFW and RDW, g per plant) and relative water content (RWC, %) were evaluated on the same plants. In the fine-mapping experiments (E4–E7), rootstocks were dissected. The number of seminal roots (N.SR, count) and the number of whorls where shoot-borne roots originated were counted (N.whorls, count). The number of shoot-borne roots reaching the soil was counted for all developed whorls separately and aggregated (N.SBR, count). From whorl 2 to whorl 7, three randomly selected excised roots per whorl were photographed with a black background, analysed with the software DIRT for the excised root traits, and visually scored for LR length.

The collected shoot traits included stand count (STC, count), early vigour score (EV, 1–9 score), plant height (PH, cm), leaf greenness measured with a chlorophyll content meter (SPAD-502Plus, Konica Minolta, Inc., Japan) (SPAD), male and female flowering (d after sowing), anthesis–silking interval (ASI, d), shoot fresh and dry weight (SFW and SDW, g per plant), shoot water content (SWC, %), grain fresh and dry yield (GFY and GDY, dt ha^–1^), and grain dry matter content (GDC, %). SFW, SDW, and SWC were measured on the same plants harvested for the root trait assessments. GFY, GDY, and GDC were determined from the remaining plants in each plot at the R6 stage. The other measurements in the field experiments were taken on three randomly chosen plants per plot. Scores and counts refer to the entire plot. In the greenhouse, measurements were taken on single plants. For detailed information on trait abbreviations and assessment in different experiments, refer to Supplementary Table S3.

### Phenotypic data analysis

All statistical analyses were conducted in R (R Core Team, 2025). The statistical model used for estimating genotype and genotype by trial variance components was:


(1) $$y_{\mathrm{lemjk}}=\mu +\eta_{l}+\gamma_{e}t_{m}+g_{j(l)}+\gamma_{e}tg_{\mathrm{mj}(l)}+b_{k(m)}+e_{\mathrm{lemjk}}$$

Where $y_{\mathrm{lemjk}}$ are the phenotypic observations, μ is the overall mean, $\eta_{l}$ is the effect of the group (l=1,2 denotes checks and genotypes from the bi-parental cross, respectively), $\gamma_{e}$ is a dummy variable with $\gamma_{e}=1$ in analyses comprising experiments with several trials (E0, E3, E4, and E5) and $\gamma_{e}$=0 otherwise, $t_{m}$ is the effect of the trial m, $g_{j(l)}$ is the effect of genotype j nested in group l, $tg_{\mathrm{mj}(l)}$ is the interaction of trial m and genotype j nested in group l, $b_{k(m)}$ is the effect of block k nested in trial m, and $e_{\mathrm{lemjk}}$ denotes the residual error. Phenotypic observations from plots with fewer than three plants were set to missing values. Outliers were manually curated by inspection of the residual plots.

To estimate variance components, $g_{j(l)}$was treated as fixed for l=1; all other effects except $\eta_{l}$ were treated as random and were assumed to be independent and identically normally distributed with mean zero and the respective variance component. Variance components and their SEs were estimated using the restricted maximum-likelihood method implemented in ASReml-R package version 4 (Butler *et al.*, 2017).

Trait heritabilities (*h*^2^) were calculated on an entry-mean basis according to Holland *et al.* (2002). Variance component estimates were considered significant when exceeding twice their standard errors.

Adjusted entry means within and across trials were obtained from Equation 1, treating $g_{j(l)}$ and $t_{m}$ as fixed effects and dropping the term $\eta_{l}$ from the model. The significance of the fixed effects was tested with an incremental Wald test implemented in ASReml-R. Fixed terms were considered significant for *P*<0.05.

Phenotypic correlations among traits were calculated as the Pearson correlation coefficient of the adjusted means within and across trials for pairwise trait combinations. To account for multiple testing, we applied the Bonferroni–Holm correction (Holm, 1979). Correlations were considered significant for *P*<0.05.

### Genotypic data analysis

The two parental lines, 550 F_2_ individuals phenotyped in E1, and 284 F_3_ founder individuals were genotyped using the 15 K SNP array. SNPs not represented in the 600 K array, with missing calls in P1 or P2, heterozygous in P1 or P2, with >5% missing calls, or with >5% non-parental alleles were discarded. Of the SNPs overlapping with the 600 K array, 97.4% and 96.7% were retained for the F_2_ and F_3_ datasets, respectively. Among the retained SNPs, 27.9% were polymorphic in the F_2_ and 17.6% in the F_3_, with 2212 overlapping between the two datasets. These overlapping SNPs were used for constructing genetic maps and for QTL mapping.

### Genetic maps and quantitative trait locus mapping

Two genetic maps were generated based on the Haldane mapping function with genotypic data from F_2_ and F_3_ individuals using the package R/qtl (Arends *et al.*, 2010). The marker order in both maps corresponded to their physical order on the B73 AGPv5 (Hufford *et al.*, 2021) physical map (Supplementary Fig. S3). One F_3_ individual with an excessive number of crossing overs was excluded from QTL mapping.

For the F_2_ QTL mapping, phenotypic data on LR length from individual F_2_ plants at stage R6 (E1) and from adjusted progeny means for LR length at stage V6 (E2) were used. The F_3_ QTL mapping was performed on adjusted progeny means from F_3:4_ progenies averaged across trials (E3) for traits exhibiting significant genetic variance. Multiple QTL mapping was conducted using the ‘stepwiseqtl’ function of the R/qtl package (Arends *et al.*, 2010), which applies forward selection (up to 10 QTLs) and backward elimination to identify the model with the highest penalized LOD score. Penalty values were derived from a 5000 iteration permutation test, with significance threshold of 0.01. For the most likely QTL model, we used the function ‘fitqtl’ to obtain the proportion of phenotypic variance explained (PVE) for individual QTLs and the full model, as well as for the estimation of additive and dominant QTL effects, by fitting all QTLs simultaneously. Approximate 95% Bayesian credible QTL intervals were calculated with the function ‘bayesint’. The proportion of genetic variance explained was obtained by dividing the PVE by the trait heritability (Schön *et al.*, 2004). For a given trait, the additive effects of the QTLs were expressed in SDs by dividing the QTL additive effects by the SD of the adjusted means of the respective trait. QTLs were considered overlapping when their distance on the F_3_ genetic map was ≤20 cM. Detailed descriptions of the R/qtl functions used are provided in Broman and Sen (2009).

### Fine mapping of *qlr1*

In the fine-mapping experiments (E4–E7), 14 KASP markers on Chr. 1 (M1–M14) positioned within the 26 Mb region comprising *qlr1* were used. The statistical model to estimate the additive effect of M1–M14 in individual experiments E4–E7 and across E4–E7 was:


(2) $$\begin{array}{rl} y_{\mathrm{lemipjk}}= & \mu +\eta_{l}+\gamma_{e}t_{m}+\varepsilon_{i}w_{p}+\lambda_{l\;}\beta_{q}x_{q}+\delta_{e}\lambda_{l}{t\beta }_{q}^{m}x_{q}+g_{j(l)}+b_{k(m)}\\ & +e_{\mathrm{lemipjk}} \end{array}$$

Where $y_{\mathrm{lemipjk}}$ are the phenotypic observations centred and scaled for each trait, μ is the overall mean, $\eta_{l}$ is the fixed effect of the group (l=1,2,3 for checks, RILs, and HIFs), $\gamma_{e}$ is a dummy variable with $\gamma_{e}=1$ in analyses comprising experiments with several trials (E4, E5, and across E4–E7) and $\gamma_{e}$=0 otherwise, $t_{m}$ is the fixed effect of the trial m, $\varepsilon_{i}$ is a dummy variable with $\varepsilon_{i}$=1 for root traits assessed on separate whorls and $\varepsilon_{i}$=0 otherwise, $w_{p}$ is the fixed effect of the whorl *p* (p=1–6 for whorls W2–W7), $\lambda_{l}$ is a dummy variable with $\lambda_{l}$=1 for HIFs and $\lambda_{l}$=0 otherwise, $\beta_{q}$ is the fixed additive effect of the tested marker q, $x_{q}$ is the genotype score with $x_{q}\in$ [0,1,2] for genotypes AA–AB–BB (with AA being the genotype of P1, BB of P2, and AB the heterozygous genotype), $\delta_{e}$ is a dummy variable with $\delta_{e}=1$ in analyses of experiments E4 and E5, each comprising two trials, and $\delta_{e}$=0 otherwise, ${t\beta }_{q}^{m}$ is the fixed interaction of the additive effect of the tested marker q and the trial m, $g_{j(l)}\;$is the random effect of the genotype j nested in group l, $b_{k(m)}$is the random effect of block k nested in trial m, and $e_{\mathrm{lemipjk}}$is the residual error. Random effects were assumed to be independent and identically normally distributed with mean zero and the respective variance component. The significance of the fixed effects was tested with an incremental Wald test implemented in ASReml-R. Fixed terms were considered significant for *P*<0.05.

### Genomic resources and candidate gene analysis

Parental lines P1 and P2 were sequenced using PacBio HiFi long reads by CNRGV, INRAE Occitanie, Toulouse, France (cnrgv.toulouse.inrae.fr). Circular consensus sequences were *de novo* assembled with hifiasm version 19 (Cheng *et al.*, 2021) using default settings. Protein-coding gene sequences were detected using the MAKER toolset (Campbell *et al.*, 2014), with cDNA and protein evidence based on other publicly available data. We used liftoff (Shumate and Salzberg, 2021) to map the B73v5 annotated genes within the fine-mapped region of *qlr1* (M4–M10) against the assemblies of P1 and P2, determining the respective genomic coordinates. Multiple sequence alignments of gene and protein sequences of B73, P1, and P2 were performed with the ‘msa’ package in R (Bodenhofer *et al.*, 2015) and manually inspected. The genomic sequence of the annotated genes and respective subfeatures for B73, their functional annotation, Gene Ontology terms, and expression level in root tissues of B73 referring to Stelpflug *et al.* (2016) were retrieved from the maize GDB (www.maizegdb.org) and refer to the latest annotation (Hufford *et al.*, 2021).

### Transcript level measurements

In E7, root tissues were collected from five biological replicates of eight genotypes (P1, P2, HIF6A, HIF6B, HIF7A, and HIF7B) segregating for different fragments of *qlr1* to quantify transcript abundance of candidate genes (Supplementary Fig. S4). To minimize potential effects of stem severing on gene expression, roots were washed while plants were still intact, and the stem was severed only after root tissues were harvested. To ensure comparable tissue age across genotypes, the last fully developed whorl at developmental stage R2 was chosen as the reference point, irrespective of the total number of whorls. For each plant, a 10 cm distal segment of one representative shoot-borne root from each of the last two developed whorls, including the root tip, was excised and flash-frozen in liquid nitrogen (Supplementary Fig. S4A). RNA was then extracted using a guanidine hydrochloride protocol (Logemann *et al.*, 1987), followed by DNase digestion (DNase I, RNase free, Thermo Scientific) and first-strand cDNA synthesis (Maxima H Minus Kit, random hexamer primers, Thermo Scientific K1652). Quantitative reverse transcription–PCR (RT–qPCR) was performed in three technical replicates with primers binding to the 3′-untranslated region (UTR) of the candidate genes Zm00001eb015500 (*hb130*), Zm00001eb015210 (*cipk3*), and Zm00001eb015390 (*IDP7785*). The genes MEP (membrane protein PB1A10.07c, Zm00001eb257640) and LUG (Leunig, Zm00001eb357900) were used as references (Manoli *et al.*, 2012). Normalization of RT–qPCR was performed with the ΔΔCq method (Livak and Schmittgen, 2001). We developed PCR assays to identify the alleles of *hb130* and *cipk3* in ambiguous HIFs (Supplementary Fig. S4B). To discriminate the alleles of *hb130*, differing for a tandem insertion in the intron, we designed intron-spanning primers and determined the amplicon lengths by gel electrophoresis of the PCR products. To discriminate the alleles of *cipk3*, differing for a few sequence polymorphisms, we designed primers binding to the 3'-UTR and Sanger-sequenced the PCR product. The sequences of all primers used are listed in Supplementary Table S4.

### Haplotype frequency in breeding lines

To assess the frequency of *qlr1* haplotypes in genetically improved maize inbred lines, we obtained the genotypic data of 65 flint inbred lines genotyped with the 600k array described in Unterseer *et al.* (2016). These data were merged with those of 501 DH lines from the landrace KE, retaining SNPs shared between the two datasets. To enable comparisons between landrace and breeding material, we defined haplotypes by concatenating 10 consecutive SNPs, following the approach of Mayer *et al.* (2020). Accordingly, the *qlr1* haplotype comprised 10 SNPs, including the most significant SNP associated with LR length (M8, AX-90529242). Haplotype alleles occurring less than three times in the combined set of genotypes were excluded.

## Results

### Heritability of root and agronomic traits

Two DH lines derived from the Austrian maize landrace ‘Kemater Landmais Gelb’, KE0413 (P1) and KE0113 (P2), differed significantly in LR length assessed by visual scoring (LRv) at developmental stage R2 (E0), representing the extremes for this trait, while showing similar flowering time and sharing a similar genomic background. By crossing P1 and P2, we developed the populations evaluated in seven field and greenhouse experiments in this study (Fig. 1; Table 1).

LRv exhibited intermediate to high heritabilities (*h*^2^) in the DH lines (E0, *h*^2^=0.87) and in the populations derived from the bi-parental cross, ranging from 0.43 (E2, stage V6) to 0.74 (E3, stage R2) (Table 1). In E3, both average LR length (LRd) and branching frequency (LRBFd) were significantly correlated with LRv (Supplementary Fig. S5). Consequently, we considered LRv as a proxy trait for total LR length, as it integrated both components of LR architecture. LRd showed high heritability (*h*^2^=0.61), whereas LRBFd exhibited a substantially lower value (*h*^2^=0.33). RDW, flowering time (FF), early plant height (PH_V6), final plant height (PH_R2), and SDW also exhibited intermediate to high heritabilities (*h*^2^=0.65–0.88) in the bi-parental populations.

### Mapping quantitative trait loci for root and agronomic traits

QTLs were detected for all traits except LRBFd and SDW_R2 (Table 2) and were distributed on all chromosomes, with some QTLs co-localizing in the same genomic regions (Fig. 2). For LRv, eight QTLs were detected at developmental stage R2, five at R6, and one QTL at V6, explaining 57, 24, and 13% of the phenotypic variance, respectively. All five QTLs for LRv at stage R6 overlapped with the respective QTLs at stage R2 even though QTL mapping was performed in separate experiments (E1 and E3). The QTLs on Chr. 7 at stage V6 did not overlap with any of the QTLs at reproductive stages. The developmental stage R2 was chosen as the reference point to assess root traits in all subsequent experiments because of the higher heritability and number of QTLs detected for LRv. Notably, the LRv QTLs on Chr. 1 (53 Mb) co-localized with a QTL for LRd. The LRv QTLs on Chr. 8 (107 Mb) and Chr. 9 (163 Mb) co-localized with QTLs for RDW and FF, respectively, while the other LRv QTLs were not associated with other traits.

**Table 2.** QTLs identified for traits with significant genetic variance in experiments E1–E3

Trait	*h* ^2^	Chr	Pos (Mb)	AE±SE	PVE (%)	PVEm (%)	GVE (%)	GVEm (%)
LRv_R2	0.74	1	56	−0.28±0.04	9.2	57.4	12.4	77.6
		3	187	−0.29±0.04	8.9		12.0	
		5	6	−0.25±0.04	6.4		8.7	
		5	194	0.24±0.04	7.3		9.8	
		8	20	−0.23±0.04	5.1		6.8	
		8	107	−0.25±0.04	5.5		7.4	
		8	164	0.23±0.04	6.4		8.6	
		9	163	−0.21±0.04	4.6		6.3	
LRv_R6		1	52	−0.47±0.08	5.3	24.2		
		3	191	−0.39±0.08	3.0			
		5	13	−0.40±0.09	4.3			
		5	206	0.62±0.08	7.9			
		8	17	−0.43±0.08	3.7			
LRv_V6	0.43	7	19	−0.31±0.07	13.3	13.3	30.8	30.8
LRd_R2	0.61	1	54	−1.76±0.29	10.9	18.5	17.8	30.3
		10	151	−1.41±0.31	7.9		13.0	
RDW_R2	0.65	6	176	−2.99±0.55	9.4	20.4	14.4	31.5
		8	109	−3.15±0.53	10.3		15.9	
FF	0.88	1	22	−0.87±0.13	6.9	58.5	7.9	66.5
		2	3	0.80±0.14	6.2		7.0	
		3	5	0.68±0.13	4.2		4.8	
		7	2	0.64±0.13	3.9		4.5	
		7	185	−0.74±0.13	4.9		5.6	
		9	160	−1.18±0.13	12.1		13.7	
		10	151	1.31±0.13	18.3		20.8	
PH_V6	0.75	10	151	−3.88±0.36	31.9	31.9	42.6	42.6
PH_R2	0.71	6	158	3.51±0.59	10.9	19.4	15.3	27.3
		8	142	−3.37±0.58	10.2		14.3	

Given are the trait heritability (*h*^2^), chromosome (Chr), and physical position in megabases of the QTLs [Pos (Mb)], estimated additive effect of the QTL P2 allele ±SE (AE±SE), proportion of phenotypic and genetic variance explained by the individual QTL [PVE (%), GVE (%)] and by the multi-QTL model [PVEm (%), GVEm (%)].

Trait codes: LRv, visual scoring of LR length (at stages V6, R2, R6); LRd, DIRT assessment of LR length (at stage R2), FF, flowering time; PH plant height (at stages V6, R2), RDW root dry weight (at stage R2).

The additive effects of the LRv QTLs at stage R2 were moderate, ranging from scores of 0.21 to 0.29 (corresponding to 0.25–0.35 SDs of the trait). At six of the eight LRv QTLs, the trait increasing alleles were contributed by P1 (Table 2), explaining the large phenotypic difference between parental lines despite the moderate effect sizes of single QTLs. The QTL on Chr. 1, explaining the highest proportion of phenotypic variance for both LRv and LRd, was targeted for fine-mapping and will subsequently be referred to as *qlr1*.

### Assessing lateral root length in different whorls

**Fig. 3.**
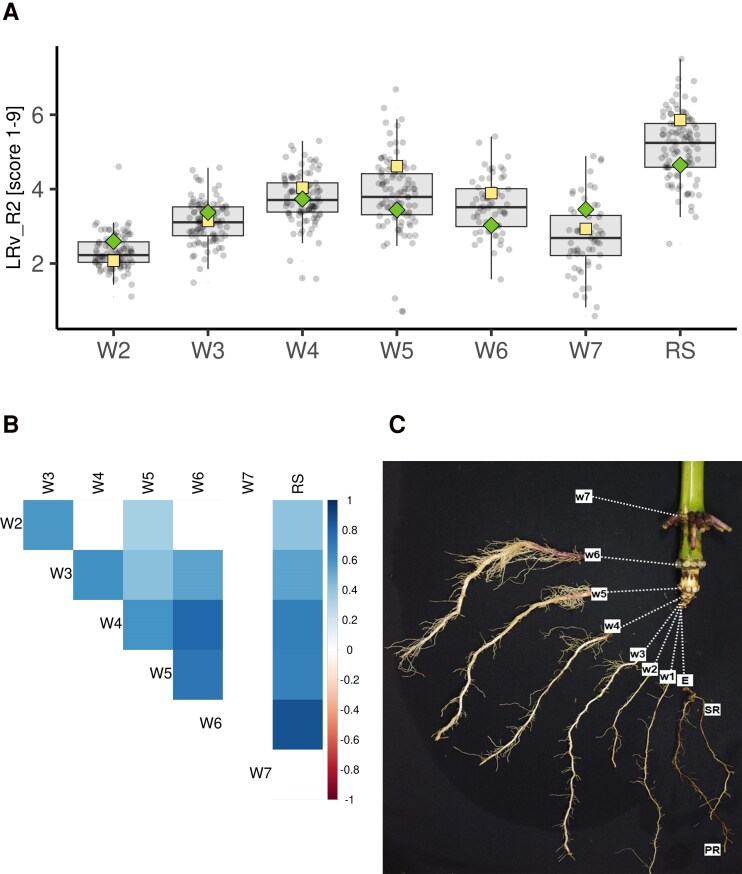
Assessment of visually scored lateral root (LR) length in different whorls at developmental stage R2 (LRv_R2). (A) Distributions of LRv_R2 measured on the rootstock (RS) and excised roots of whorls W2–W7 of 91 HIF progenies, four RILs, and five checks. Dots represent adjusted means across experiments (E4–E7, *n*=100). P1 and P2 are shown as squares and diamonds, respectively. Boxplots show medians (thick lines), first and third quartiles (hinges), and whiskers extending to 1.5 times the interquartile range. (B) Pairwise Pearson correlations of LR length measured from the RS and W2–W7. Only significant correlations (*P*<0.05, Bonferroni–Holm-corrected) are displayed. (C) Root system dissection of a representative plant from this study at stage R2. Embryonic roots (E), including the primary root (PR) and seminal roots (SR), and shoot-borne roots of whorls W1–W7 are visible.

In the fine-mapping experiments comprising 43 HIFs segregating for the *qlr1* QTL (E4–E7), we dissected the root system and measured LRv, LRd, and LRBFd on separate whorls (W), from W2 to W7. The shoot-borne roots accessible at stage R2 capture the developmental history of the root system, with older whorls still being accessible and no new ones emerging, as the root system reached full maturity. Analogously to our findings on the rootstock (Supplementary Fig. S5), LRv on whorl-specific excised roots correlated strongly with LRd (Supplementary Fig. S6), with the former displaying slightly higher heritability (Supplementary Fig. S7). LRv was also weakly to moderately correlated with LRBFd. However, LRBFd displayed low heritability. Across trials and genotypes, LRv increased from W2 to W5, decreased from W5 to W7, and showed intermediate to high positive correlations between whorls (Fig. 3). LRv in W2, W3, and W4 was only weakly correlated with the number of whorls and of shoot-borne roots (Supplementary Fig. S8), suggesting minimal competition among these components of root architecture.

### Fine-mapping *qlr1*

**Fig. 4.**
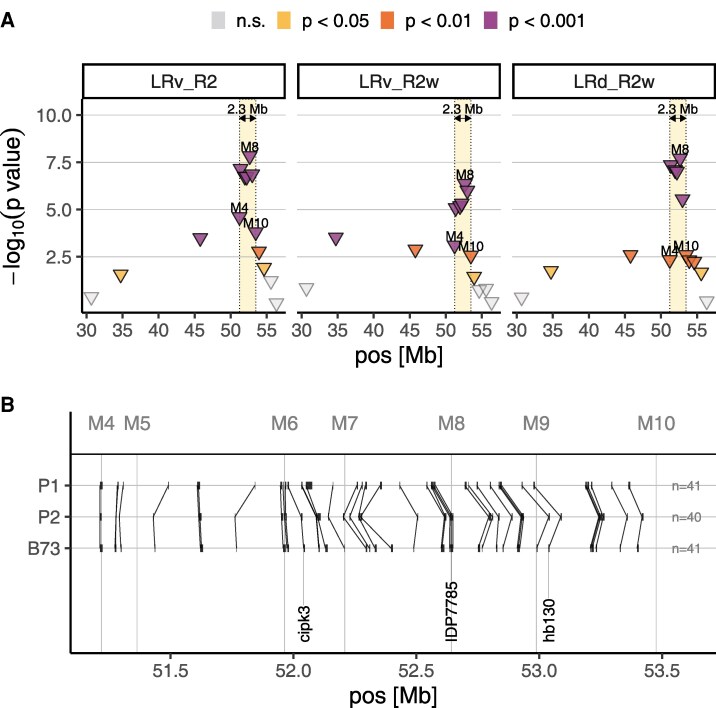
Fine mapping of QTL *qlr1*. (A) Significance of the marker–trait associations of 14 markers located within *qlr1* with lateral root (LR) length at developmental stage R2 across experiments (E4–E7). LR length was assessed with a visual scoring on the rootstock (LRv_R2) and averaged across excised roots from W2 to W7 with visual scoring (LRv_R2w) and with DIRT (LRd_R2w). Markers are represented by triangles and coloured according to the level of significance of the marker–trait associations. The fine-mapped region (M4–M10) is indicated by a dotted box. Flanking markers M4 and M10 and the most significant marker M8 are labelled. The *x*-axis represents the physical position on chromosome 1 based on B73v5 in megabases. (B) Annotated genes between M4 and M10 in P1 (KE0413), P2 (KE0113), and B73 (B73v5). Genes are represented as vertical segments in the respective genomic positions and connected if syntenic. For visualization on a common axis, the starting coordinate of the first gene of B73 is taken as reference for all genomes. Marker positions are based on B73v5. Three candidate genes are labelled: *hb130*, *cipk3*, and *IDP7785*.

Marker–trait associations detected for LRv and LRd across experiments E4–E7 pointed consistently to the region between markers M5 (AX-90529220, pos. 51 364 010) and M9 (AX-90529237, pos. 52 988 306) (Fig. 4A).

The significance of marker–trait associations dropped sharply for flanking markers M4 (AX-91366105, pos. 51 218 801) and M10 (AX-91334117, pos. 53 476 179), thus identifying a 2.3 Mb interval on Chr. 1 delimited by M4 and M10 to harbour candidate genes for *qlr1*. The 2.3 Mb region contained 46 protein-coding gene models annotated in B73v5, of which 41 and 40 were syntenic in P1 and P2, respectively (Fig. 4B). We examined the expression of these genes in B73 shoot-borne roots, polymorphisms between P1 and P2, functional annotation, and literature evidence to prioritize candidate genes (Supplementary Table S5). Twenty-five genes were expressed in the crown roots of B73 and were polymorphic at the gene sequence level between P1 and P2, of which 17 had polymorphisms in their predicted protein sequences. Three genes emerged as potential candidates from functional annotation and literature evidence: Zm00001eb015500 (*hb130*), Zm00001eb015210 (*cipk3*), and Zm00001eb015390 (*IDP7785*). *hb130* encodes a homeobox transcription factor functionally annotated for adventitious root development and LR formation. P1 and P2 alleles of *hb130* differed for SNPs in the UTRs, a tandem insertion in the first intron, and several polymorphisms in the coding sequence resulting in two amino acid substitutions and a small insertion in the protein product (of 314 amino acids). *cipk3* encodes a calcineurin B-like-interacting protein kinase associated with seminal root length and drought tolerance in maize (Li *et al.*, 2023). P1 and P2 alleles of *cipk3* were polymorphic in the UTRs but encode identical proteins (of 443 amino acids). *IDP7785* codes for an ADP-ribosylation factor, was reported as a GWAS candidate gene for primary root length in maize seedlings (Zuffo *et al.*, 2022), and differed for several polymorphisms in the 5'-UTR and coding sequence, resulting in nine amino acid substitutions and a deletion of four amino acids in the protein product (of 653 amino acids). In E7, we measured the transcript abundance of the three prioritized candidate genes in two HIFs (HIF6 and HIF7) and parental lines (Supplementary Fig. S4). The alleles of all candidate genes were segregating in HIF6, while the P1 allele of *cipk3* was fixed in HIF7. *hb130* exhibited significant allele-specific differences both between the parental lines and in one segregating HIF, whereas for *cipk3* and *IDP7785*, significant differences were observed only between the parental lines (Supplementary Fig. S9).

### Characterizing *qlr1*

**Fig. 5.**
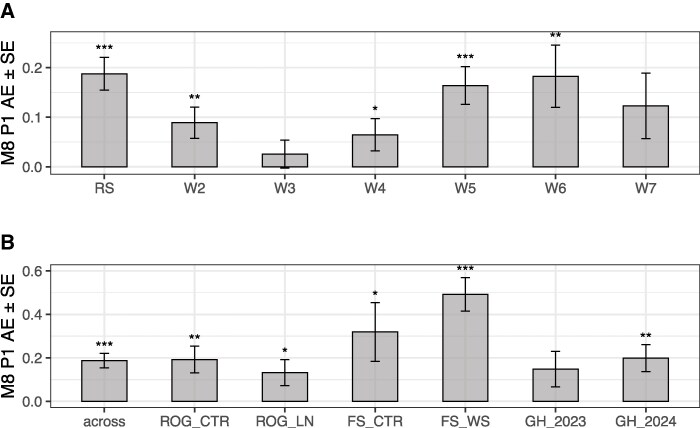
*qlr1* characterization. Additive effects of the P1 allele at QTL *qlr1* estimated based on the association of marker M8 with the visual scoring of lateral root length (LRv) at developmental stage R2 in different whorls (A) and experiments (B). (A) Allele effects for LRv measured on the rootstock (RS) and separate whorls (W2–W7) on 43 HIFs across experiments E4–E7. (B) Allele effects for LRv on the RS across experiments E4–E7 and in individual trials. Estimations are based on 43 HIFs across experiments, 25 HIFs in ROG, 3 HIFs in FS, 13 HIFs in GH_2023, and 9 HIFs in GH_2024. Bars represent the additive effect ±SE (AE±SE) of the P1 allele estimated at marker M8, expressed as SDs of the respective trait. Significance of the allele effects is marked with asterisks: ***P<0.001, **P<0.01, *P<0.05. Trial abbreviations: ROG, Roggenstein; FS, Freising; CTR, control; LN, low N; WS, water stress; GH, greenhouse; 2023, 2024 year.

Marker M8 (AX-90529242, pos. 52 643 392) consistently showed the highest significance across experiments and was selected as the representative marker for *qlr1*. In the 43 HIFs, the P1 allele of M8 was significantly associated with increased LRv and LRd when assessed both on the RS and on individual whorls (Fig. 5A). Across E4–E7, the *qlr1* effect was significant in all whorls except for W3, and was most pronounced in later forming whorls W5 and W6. The *qlr1* additive effect of 0.23–0.25 scores for LRv on the RS, W5, and W6, was in line with the results from the QTL mapping experiment (E3). *qlr1* impacted LRv and LRd significantly in all experiments (E4–E7), albeit effect sizes varied across environments and trials (Fig. 5B). Significant marker associations (*P*<0.001) were also detected for plant height at stages V6 and R2 (PH_V6, PH_R2), with the P1 allele increasing trait expression in all cases.

We identified seven haplotypes at the *qlr1* locus in the combined panel of 501 DH lines derived from the KE landrace and 65 improved flint lines. The *qlr1* haplotype derived from parent P1 was the most common in the improved flint lines (31%), and the second most common in KE DH lines (26%). Conversely, the *qlr1* haplotype derived from parent P2 was the most common in KE (51%) and occurred at intermediate frequency in the improved lines (18%) (Supplementary Fig. S10).

### Correlating shoot weight and lateral root length under different N, P, and water treatments

**Fig. 6.**
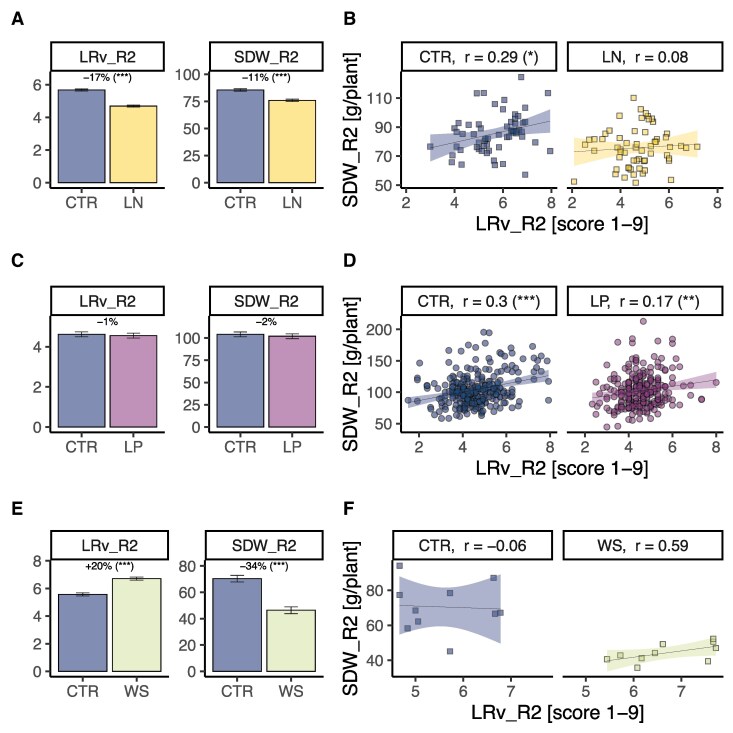
Functional consequences of lateral root (LR) length. Effect of nutrient and water treatments on visually scored LR length on the rootstock (LRv_R2) and shoot dry weight (SDW_R2) as well as their correlation. (A, C, E) Average LRv_R2 and SDW_R2 under control and treatment conditions. Bars represent the adjusted treatment means ±SE. The treatment effect, expressed as a percentage relative to control, along with its significance, is indicated above the bars. (B, D, F) Scatterplot of LR length (*x*-axis) and SDW (*y*-axis). Dots represent adjusted means within trials. The Pearson correlation coefficient and its significance are shown above each scatterplot. The regression line and its associated confidence interval (shaded area) were fitted with the function ‘geom_smooth’ in ggplot2. Underlying data came from experiment E4 (A, B), E3 (C, D), and E5 (E, F). For data dimensions, see Table 1. Significant differences are marked with asterisks: ***P<0.001, **P<0.01, *P<0.05. Treatment abbreviations: CTR, control; LP, low phosphorus; LN, low nitrogen; WS, water stress.

In E3–E5, we evaluated RILs, HIFs segregating for *qlr1*, flint maize inbred lines, and the parents of the mapping populations under control conditions and limited P, N, and water availability, detecting significant treatment effects on both root and shoot traits (Supplementary Table S6). The average response of LRv and SDW, as well as the correlation between these traits under different N, P, and irrigation treatments at developmental stage R2 is shown in Fig. 6. Under N-limited conditions (E4), both LRv and SDW were significantly reduced (*P*<0.001) compared with the control treatment (Fig. 6A). The two traits were significantly correlated under control conditions (*r*=0.29, *P*<0.05), but not under N stress (Fig. 6B). The P-limited conditions (E3) had no effect either on LRv or on SDW compared with the control (Fig. 6C), and the correlations between the two traits were significant but small in both treatments (*r*≤0.30) (Fig. 6D).

Under limited water supply (E5), mean LRv was significantly increased compared with control conditions (*P*<0.001), while mean SDW was significantly reduced (*P*<0.001) (Fig. 6E). No significant correlation could be observed between the two traits in either of the two treatments, most probably due to the small sample size (*n*=10) (Fig. 6F).

## Discussion

### Deciphering the genetic basis of lateral root length in maize

Adapting the root architecture to target environments can enhance production stability and sustainability of our crops. Breeding maize (*Zea mays* L.) varieties with this goal in mind necessitates a comprehensive understanding of the genetic regulation of root traits and their functional consequences on yield, yield stability, and resource use efficiency. Among root traits, LR architecture is of prime importance (Lynch, 2018, 2022). In this study, we leveraged the native diversity of an Austrian maize landrace to understand the genetic basis of LR length and other root and shoot traits across the life cycle of the maize plant (Fig. 1).

Our knowledge of the genetic control of LR development on adult, shoot-borne roots in maize is limited mainly due to the lack of efficient phenotyping methods. This limitation makes it practically impossible to evaluate large numbers of plants, a key prerequisite in plant breeding. Here we showed that the visual scoring of LR length from rootstock pictures (LRv, Supplementary Fig. S2) provided a high-throughput phenotyping method to quantify total LR length, integrating both LR average length (LRd) and branching frequency (LRBFd), exhibiting high heritability (Table 1) and a strong correlation with the very time-consuming assessment of LR architecture from excised shoot-borne roots (Fig. 3).

In the populations used in this study, variation in LRv was primarily driven by differences in LR length, as indicated by its stronger correlation with LRd compared with LRBFd (Supplementary Fig. S5). This pattern was particularly evident in the fine mapping experiments E4–E7, where phenotypic variation was closely associated with the segregation of *qlr1*, a QTL affecting LRv and LRd but not LRBFd (Supplementary Fig. S6). Heritability estimates of LRv and LRd reported here exceeded previously published results for LR-related traits (Burton *et al.*, 2014; Schneider *et al.*, 2020), indicating a strong genetic component contributing to trait expression in the landrace-derived plant material. This allowed the identification of five QTLs for LRv with high consistency across experiments, developmental stages, and environments (Fig. 2). The overlap of QTLs at reproductive stages R2 and R6 and their absence at stage V6 suggests that distinct genes have influenced LR architecture at different developmental stages. However, this warrants further research as differences in plant development at harvest, population size, and growing conditions might have contributed to these differences between vegetative and reproductive stages.

All QTLs we identified for LRv and LRd had moderate to small effect sizes (Table 2), consistent with a quantitative genetic architecture, similar to what was reported for other shoot-borne root QTLs (Burton *et al.*, 2014; Z.H. Zhang *et al.*, 2018; Ren *et al.*, 2022; Li *et al.*, 2024). Except for one LRv QTL that co-localized with a QTL for flowering time, all other LRv QTLs were not associated with shoot traits, supporting their potential use in breeding programmes aimed at modifying root architecture without impacting adaptive traits.

### Fine mapping a quantitative trait locus for lateral root length in adult maize

Mapping a QTL at high genetic resolution is crucial to avoid linkage drag when the QTL is introgressed by marker-based breeding and provides the starting point for candidate gene identification and map-based cloning. The QTL *qlr1* exerted parallel effects on LRv and LRd in different whorls of shoot-borne roots across growing conditions and nutrient regimes (Fig. 5), indicating that its effect on total LR length was primarily mediated through increased LR length rather than branching frequency. The large number of HIFs tested in E4–E7 allowed us to fine-map *qlr1* to a 2.3 Mb region comprising 46 genes annotated in B73v5 (Fig. 4). Three of the 46 genes (*hb130*, *cipk3*, and *IDP7785*) were identified to have potentially causal effects on the target trait. All three candidates exhibited higher transcript abundance of the P2 compared with the P1 allele (Supplementary Fig. S9). However, only *hb130* showed a significant allele-specific difference within HIFs, in which, compared with the comparison between the parents, potential background effects can be excluded. Genes homologous to *hb130* have been shown to modify root and plant architecture in rice (*Oryza sativa*) and Arabidopsis (*Arabidopsis thaliana*), including LR formation (Sheng *et al.*, 2017), LR primordium size (Kawai *et al.*, 2022), crown root (Zhao *et al.*, 2009; Cheng *et al.*, 2016; Zhou *et al.*, 2017; T. Zhang *et al.*, 2018), and tiller formation (N. Zhang *et al.*, 2018). Overexpression of *cipk3* has been associated with longer seminal roots in maize, higher LR number in Arabidopsis, and higher survival rate under drought stress (Li *et al.*, 2023). In contrast, in our population, higher *cipk3* expression was associated with decreasing LR length. *IDP7785* has been identified as a candidate gene for primary root length in maize seedlings but was not associated with LR length (Zuffo *et al.*, 2022). Thus, we consider *hb130* as the candidate gene with the highest priority. However, based on the current evidence, we cannot exclude the possibility that other candidate genes within the region may contribute to the effect of *qlr1*. The differential expression of *hb130* is possibly linked to a tandem insertion in the first intron of the allele of P1, which is the most likely variant impacting the transcription rate and/or post-transcriptional regulation, as the gene sequence and core promoter elements are otherwise similar in P1 and P2. The involvement of *hb130* homologues in regulating root architecture suggests a complex regulatory role. For example, overexpression of *wox11*, the closest Arabidopsis orthologue of *hb130*, reduced LR density in plants grown *in vitro*. Interestingly, the repression of the same gene also led to reduced LR density, but only in soil-grown plants, suggesting context-dependent gene function (Sheng *et al.*, 2017). In maize, knockout of *hb77*, an ancient paralogue of *hb130*, decreased seminal root number while increasing LR density, suggesting pleiotropic gene action (Yu *et al.*, 2024). Thus, the role of *hb130* expression in modifying LR length in maize warrants further research.

### Assessing the functional consequences of lateral root length

We assessed the functional impact of LR length in the field by analysing root and shoot responses of genotypes varying in LR length under optimal and N-, water-, and P-limited conditions. Despite the naturally low P content of the experimental field, the absence of P fertilization had only a marginal effect on root and shoot development. The low-P trial was therefore considered a distinct environment rather than a stress treatment.

We could show that genotypes with longer LRs tended to have higher SDW in control conditions in two independent experiments (E3 and E4). Under N stress, we did not observe this effect, indicating that the association between the two traits might have been conditional on N availability (Fig. 6).

Zhan and Lynch (2015) reported a stronger relationship between LR architecture and SDW under varying N availability compared with this study. A possible explanation is that Zhan and Lynch (2015) analysed two groups of genotypes differing for LR length, branching frequency, and rooting depth. In our experiments, the phenotypic variation was continuous and mainly in LR length, and we did not observe significant competition between LR length and other aspects of root architecture. However, because roots were characterized only within the upper 25 cm of the soil profile, it was not possible to infer if rooting depth was correlated with LR length or the effects of the stress treatments on root development in deeper soil layers. Nevertheless, the correlation between LR length and SDW was either positive or not significant across all tested conditions. We therefore hypothesize that increased LR length may confer a small functional advantage across diverse field conditions by enhancing resource uptake when limitations are not too severe. Establishing a causal relationship between LR length, nutrient uptake, and plant performance will require additional evidence beyond the scope of this study.

### Evaluating the potential impact of a lateral root length quantitative trait locus for breeding

The extensive genetic diversity pertaining to landraces represents a valuable resource to promote genetic studies on root traits with potential application in breeding programmes. (Gamuyao *et al.*, 2012; Uga *et al.*, 2013). Evaluating the impact of a single QTL affecting root traits on plant productivity will require introgression of the QTL alleles into elite material, followed by extensive testing in target field conditions, encompassing different soil types, and nutrient and water regimes (Khaipho-Burch *et al.*, 2023). Marker-based selection can facilitate this task as well as the identification of additional allelic variation at *qlr1*.

The *qlr1* haplotype derived from P1 was more frequent in improved flint lines than in the KE landrace, whereas the P2-derived haplotype showed the opposite pattern (Supplementary Fig. S10). This suggests that the haplotype increasing LR length may have been favoured by selection, potentially due to the agronomic advantages conferred by longer LRs in certain environments. Because the plant material analysed in this study segregated only for the two parental alleles, we were unable to assess the effects of other *qlr1* haplotypes present in the KE landrace. Characterizing additional KE individuals or other landraces carrying alternative alleles at *qlr1* may enable the identification of superior haplotypes that could be introgressed into breeding material for germplasm improvement.

Furthermore, by screening new recombinants using the *qlr1* flanking markers and additional marker developments within the 2.3 Mb target region, fine-mapping and map-based cloning of the causal feature can be achieved. Once the causal variant is unambiguously identified, generating new variation at the *qlr1* locus in breeding material (chemical-induced mutations or genome editing) may help identify alleles with stronger effects on LR length in adult shoot-borne roots. This would enable the assessment of the isolated role of LR length on nutrient and water acquisition in maize, potentially facilitating the development of elite genotypes with enhanced nutrient uptake.

## Conclusion

We developed a highly heritable visual scoring method approximating total LR length of shoot-borne maize roots and identified several QTLs for this trait at different developmental stages. We then characterized and fine-mapped *qlr1*, the most significant QTL affecting LR length, to a 2.3 Mb region, demonstrated its stable effect across multiple growing conditions, and proposed *hb130* as the underlying candidate gene. Finally, we assessed the functional consequences of LR length, finding that genotypes with longer LRs always exhibited similar or higher SDW under different field conditions.

## Supplementary Material

erag130_Supplementary_Data

## Data Availability

All primary data to support the findings of this study are available upon request from the corresponding author. The genomic sequences of KE0113 and KE0413 underlying this article are available in the NCBI nucleotide database (https://www.ncbi.nlm.nih.gov/nuccore/) and can be accessed with accession numbers PX283072 and PX283073.

## Supplementary data

The following supplementary data are available at *JXB* online.

Fig. S1. Collection of root pictures.

Fig. S2. Scoreboards for visual scoring of lateral root length.

Fig. S3. Genetic maps used for QTL mapping projected onto the B73v5 physical map.

Fig. S4. RT–qPCR experiment design.

Fig. S5. Correlations of lateral root-related parameters.

Fig. S6. Correlation of lateral root length assessed on excised roots with visual scoring and DIRT.

Fig. S7. Heritability of lateral root length assessed on excised roots with visual scoring and DIRT.

Fig. S8. Correlations between lateral root length, number of whorls, and number of shoot-borne roots.

Fig. S9. Transcript level measurements in candidate genes.

Fig. S10. *qlr1* haplotype frequency in landraces and improved flint lines.

Table S1. Heterogeneous inbred families and recombinant inbred lines included in the fine-mapping experiments E4–E7.

Table S2. Details on the experiments presented in the study.

Table S3. List of traits assessed in experiments E0–E7 and their definitions.

Table S4. Primer pairs used in this study.

Table S5. Information on candidate genes in the fine-mapped region of the QTL *qlr1*.

Table S6. Effect of nitrogen, phosphorus, and irrigation treatments on root and agronomic traits.
