# Supplementary material for: Genetic architecture and functional consequences of lateral root length in maize
Source: J Exp Bot. 2026 Mar 12;77(14):4401–16. doi: 10.1093/jxb/erag130 (PMC13415964; doi:10.1093/jxb/erag130)
Supplement: erag130_Supplementary_Data [file erag130_supplementary_data.zip › jexbot317627-file001.pdf]

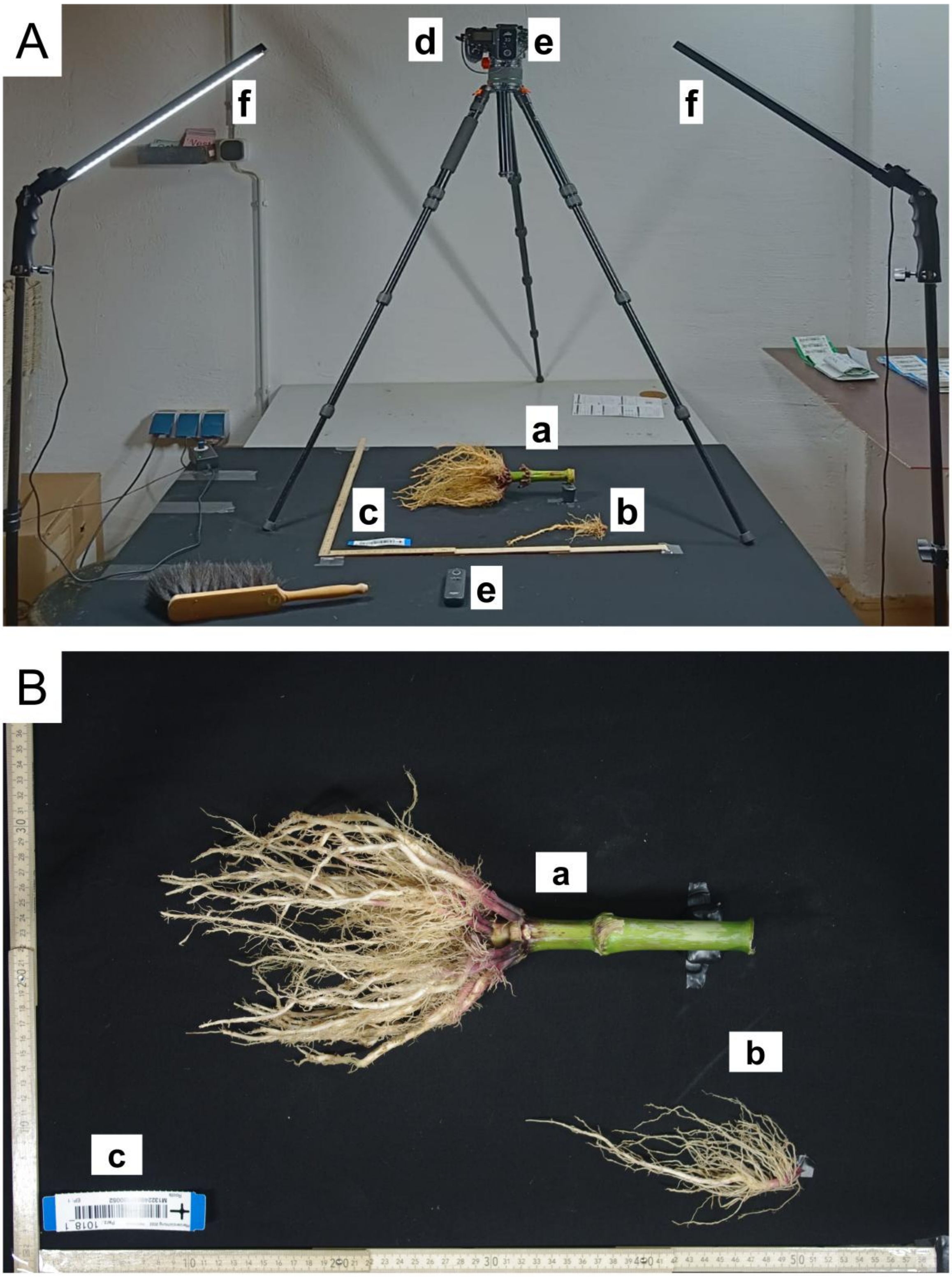

Supplementary Fig. S1. Root pictures collection.
A) Photographing station. The rootstock (a), excised root from the last developed whorl (b) and label (c) are positioned on a black cloth in the space delimited by two metre-sticks. A digital camera (d) mounted on a tripod and connected to a remote shutter (e) is used to collect the images. Artificial lights at the sides (f) ensure uniform lighting conditions.
B) Example of a rootstock picture used for root trait assessment. Rootstock and excised root were then collectively weighted to determine root biomass traits. The remaining part of the stem was weighted to determine shoot biomass traits.

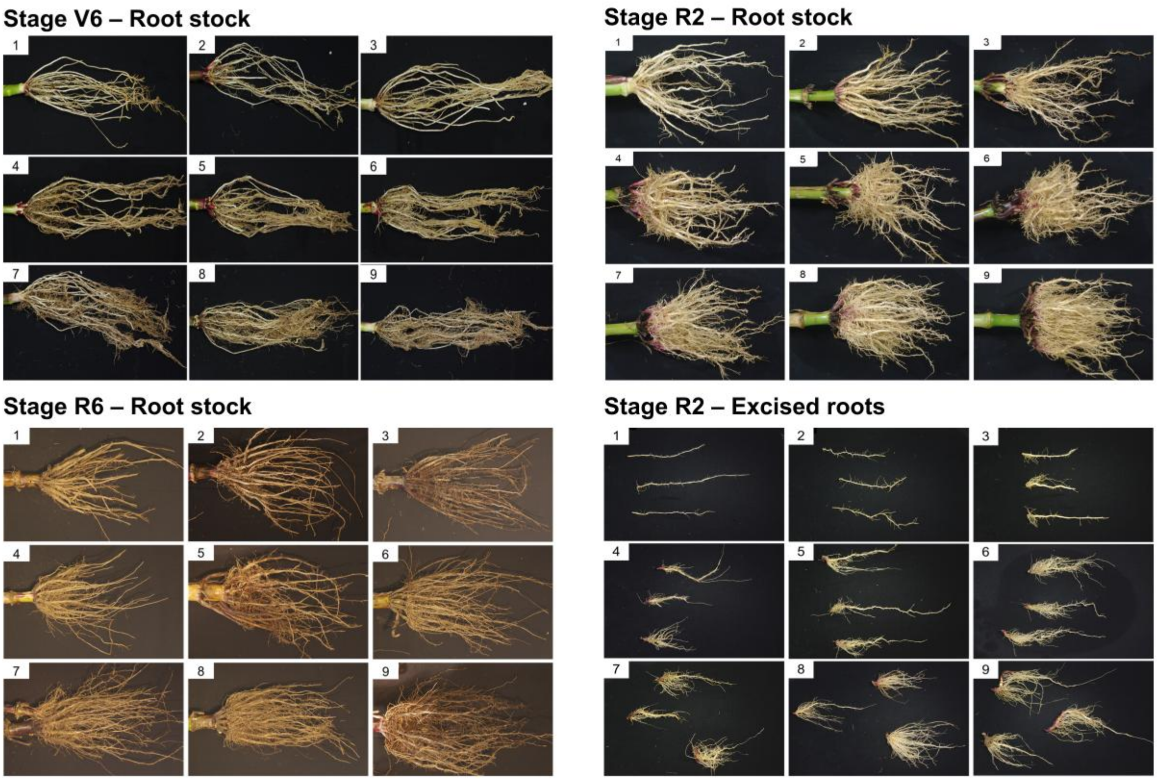

Supplementary Fig. S2. Scoreboards for visual scoring of lateral root (LR) length.
LR length was visually scored from rootstock pictures at different developmental stages (V6, R2, R6) and from pictures of excised shoot-borne roots at stage R2.

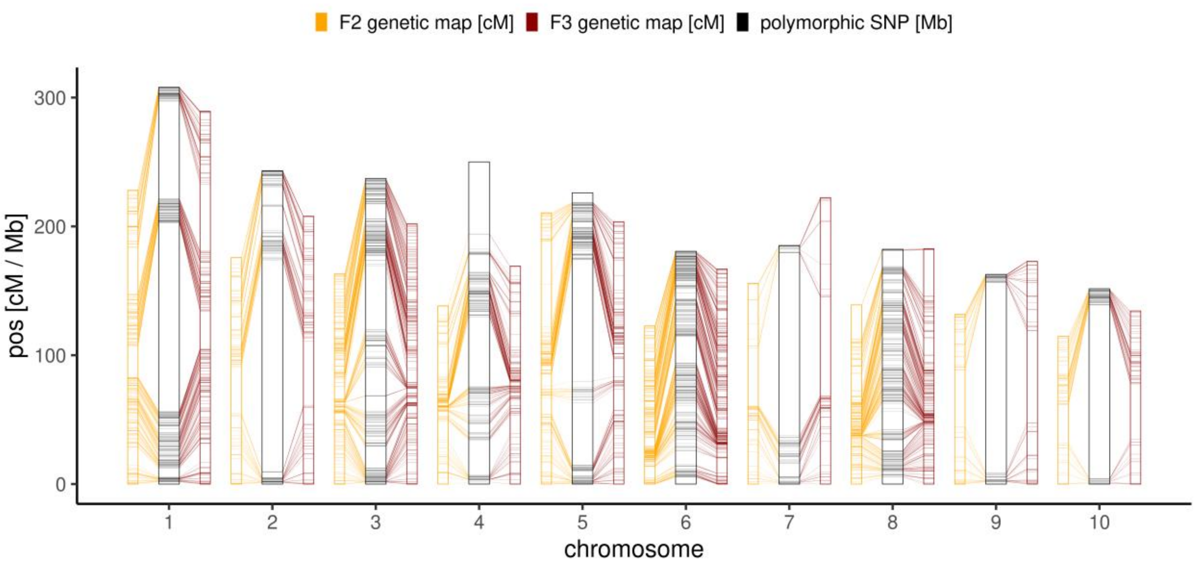

Supplementary Fig. S3. Genetic maps used for QTL mapping projected onto the B73v5 physical map.
Genetic maps were calculated from the polymorphic SNPs of 550 F2 individuals in E1 (orange, left) and of 284 F3 individuals in E3 (dark red, right). Segments connect the genetic position of the SNPs with their position onto the B73v5 physical map (black, centre).

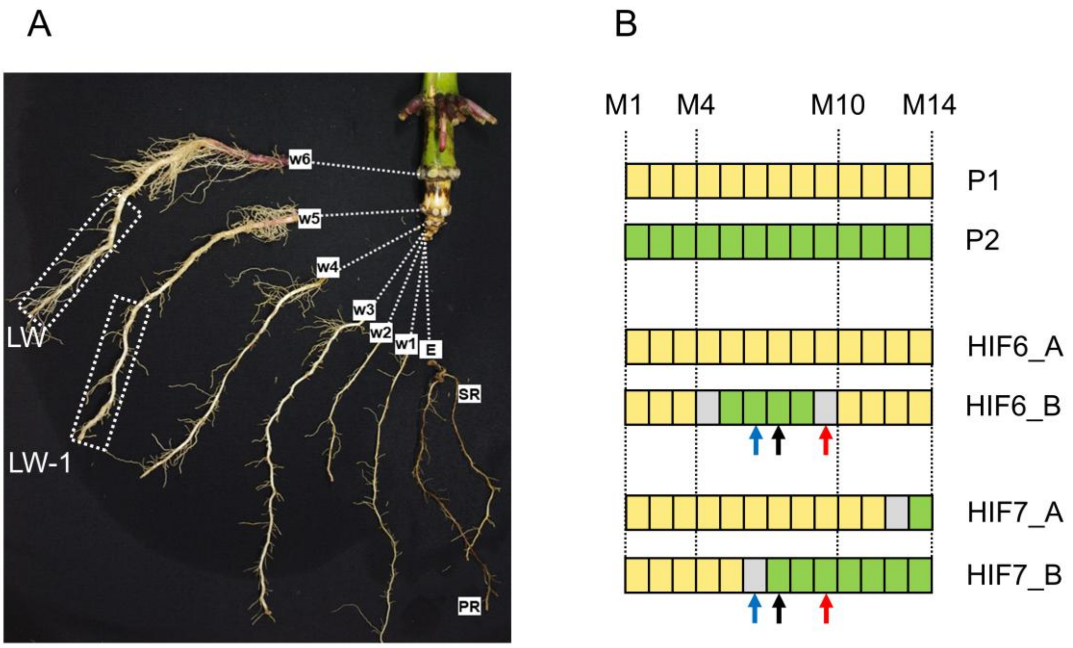

Supplementary Fig. S4. RT-qPCR experiment design.
A) Representative picture of the tissues sampled for RNA extraction, including the distal 10 cm of the last fully developed whorl (LW) and the second last fully developed whorl (LW-1).
B) Genetic composition at the *qlr1* locus of the lines included for transcript level measurements of candidate genes (P1, P2, HIF6 and HIF7). Vertical segments indicate schematically the position of the 14 KASP markers used to genotype the HIFs. The flanking markers of the genomic region and of the fine mapped *qlr1* region are labelled. Segments representing genomic sequences of P1 and P2 are coloured in yellow and green, respectively. Segments between differing marker alleles, where the recombination breakpoint is unknown, are coloured in grey. Red, blue and black arrows indicate schematically the position of the candidate genes Zm00001eb015500 (*hb130*), Zm00001eb015210 (*cipk3*), Zm00001eb015390 (*IDP7785*), respectively.

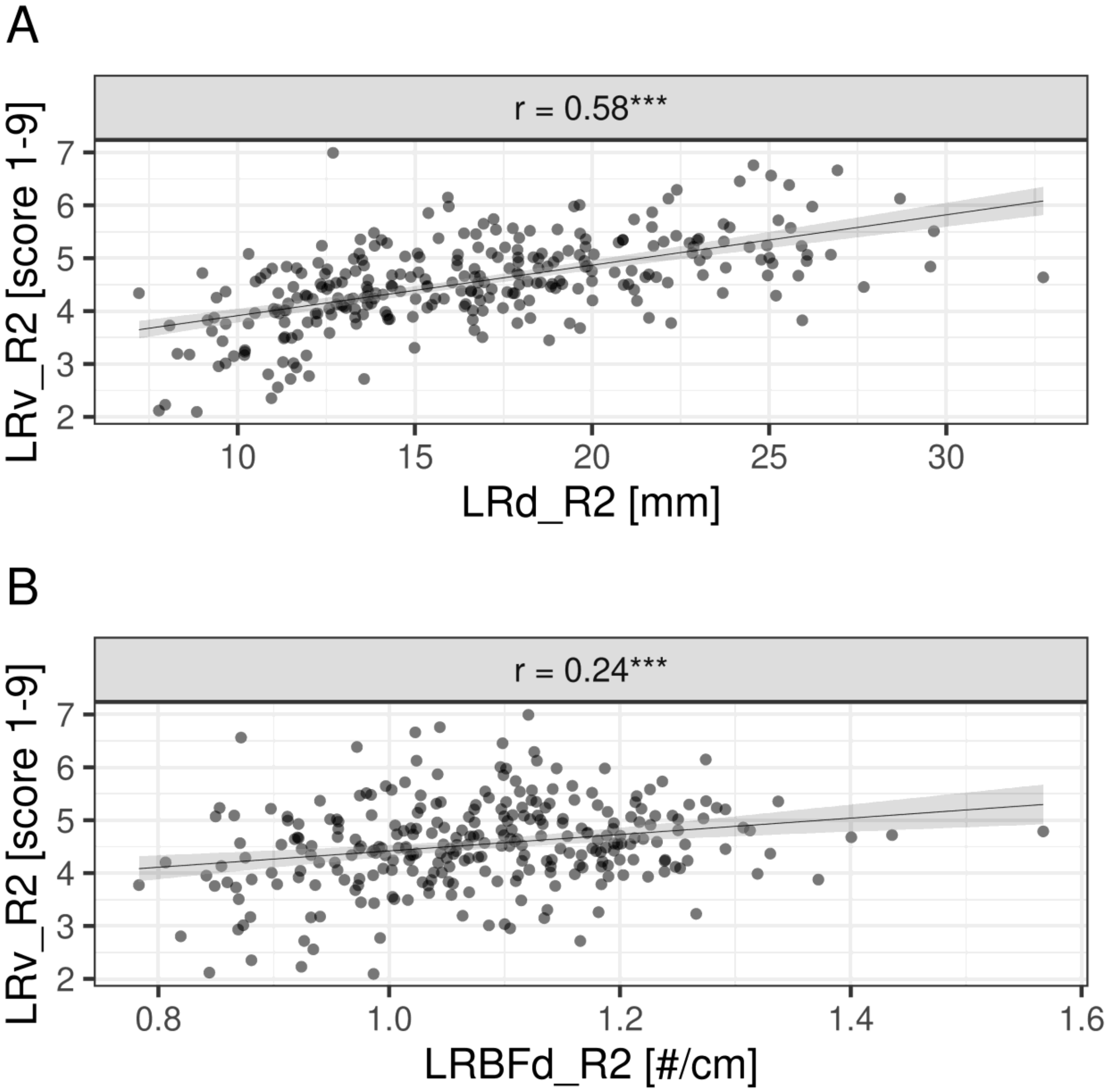

Supplementary Fig. S5. Correlations of lateral root (LR) related parameters.
A) Scatterplot of LR average length assessed with DIRT on one excised shoot-borne root from the last developed whorl (LRd_R2, x-axis) and of visually scored LR length on the rootstock (LRv_R2, y-axis) at developmental stage R2.
B) Scatterplot of LR branching frequency assessed with DIRT on one excised shoot-borne root from the last developed whorl (LRBFd_R2, x-axis) and of visually scored LR length on the rootstock (LRv_R2, y-axis) at developmental stage R2.
Individual dots represent adjusted means across trials of 290 genotypes tested in E3. The regression line and associated confidence interval (shaded area) were fitted with the function “geom_smooth” in ggplot2. The Pearson correlation coefficient (r) is displayed above the graph. Significant correlations are marked with stars: *** $P < 0.001$.

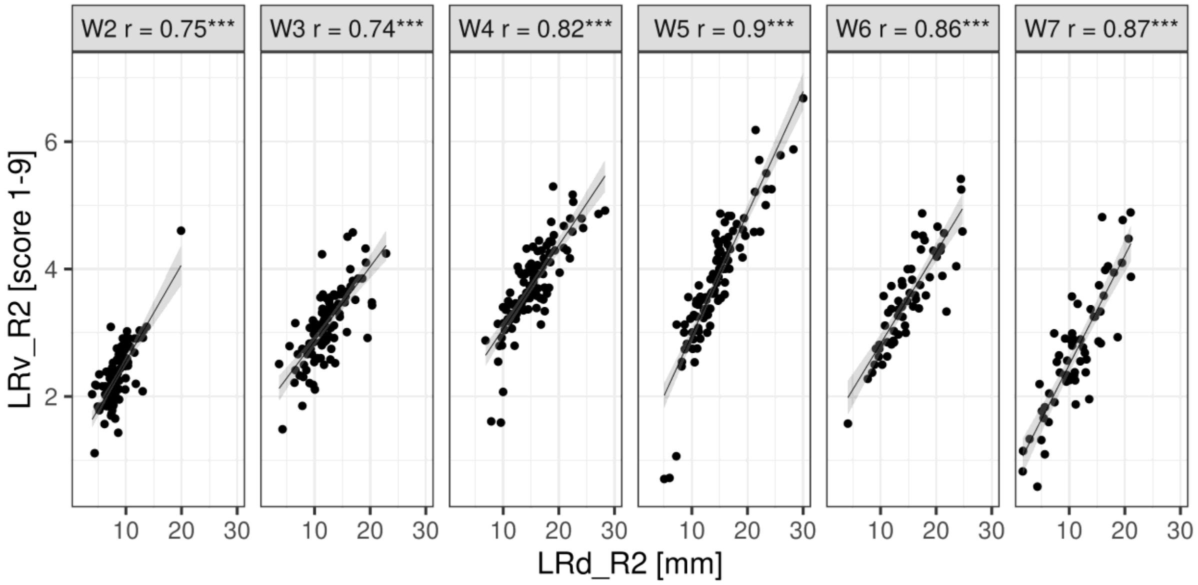

Supplementary Fig. S6. Correlation of lateral root (LR) length assessed on excised roots with visual scoring and DIRT.

Scatterplot of LR length assessed with DIRT (LRd_R2, x-axis) and with visual scoring (LRv_R2, y-axis) on three excised shoot-borne roots from different whorls (W2-W7). Individual dots represent adjusted means of 100 genotypes tested across E4-7. The regression line and the associated confidence interval (shaded area) were fitted with the function "geom_smooth" in ggplot2. The Pearson correlation coefficients (r) are displayed above the graphs ($P < 0.001$). Significant correlations are marked with stars: *** $P < 0.001$.

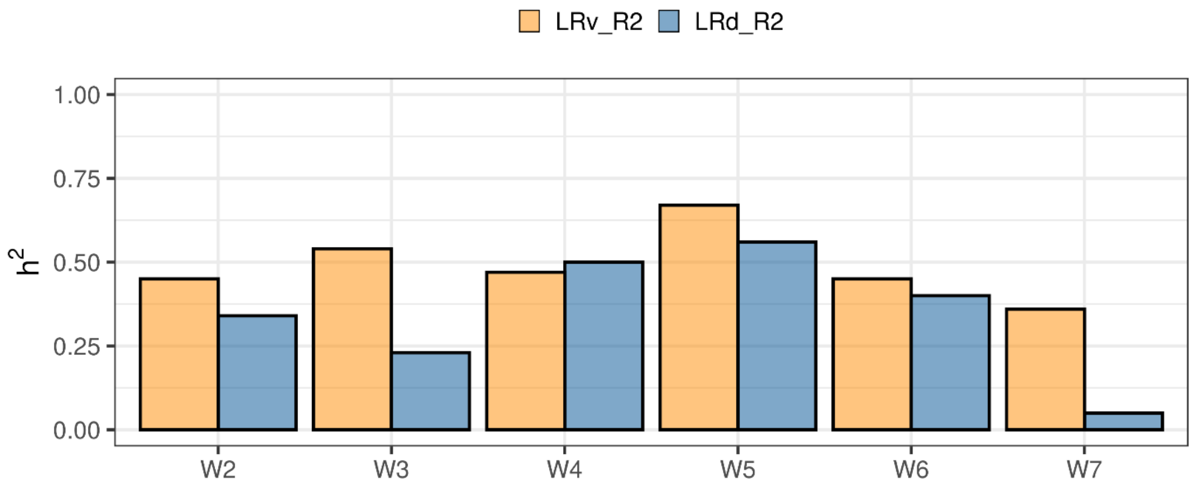

Supplementary Fig. S7. Heritability of lateral root (LR) length assessed on excised roots with visual scoring and DIRT.
Bars show heritability estimates of LR length at developmental stage R2 based on the analysis across experiments E4-7. Heritabilities of LR length are shown for the visual scoring (LRv_R2, orange) and DIRT assessment (LRd_R2, blue) in different whorls (W2-W7).

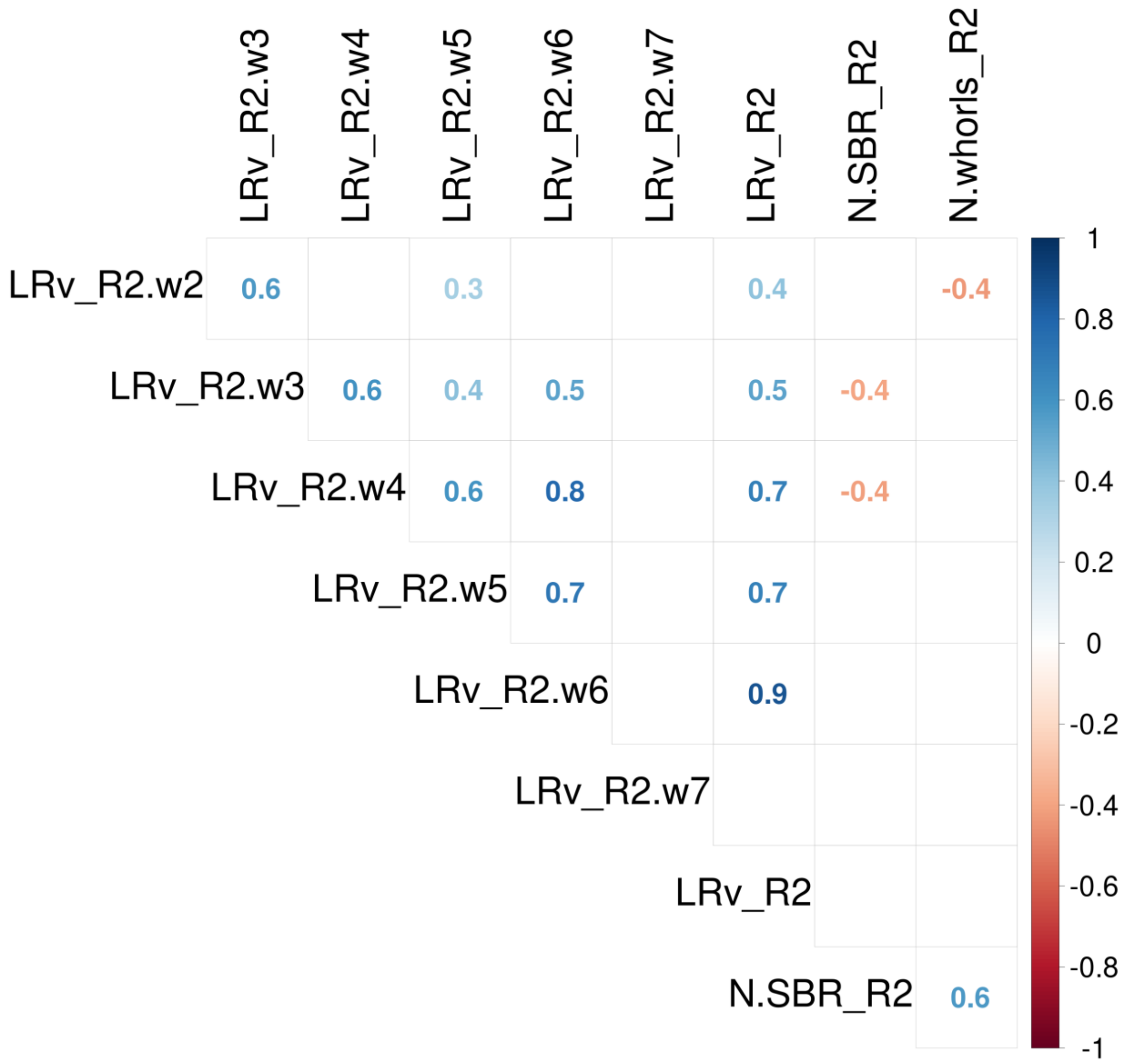

Supplementary Fig. S8. Correlations between lateral root (LR) length, number of whorls and of shoot-borne roots.
Pairwise Pearson correlations among adjusted genotype means across experiments E4-7 for visually scored LR length (LRv), number of shoot-borne roots (N.SBR) and number of whorls (N.whorls) at developmental stage R2. The suffixes w1-w7 denote traits measurements on individual whorls. Only significant correlations ($P < 0.05$, Bonferroni–Holm corrected) are displayed.

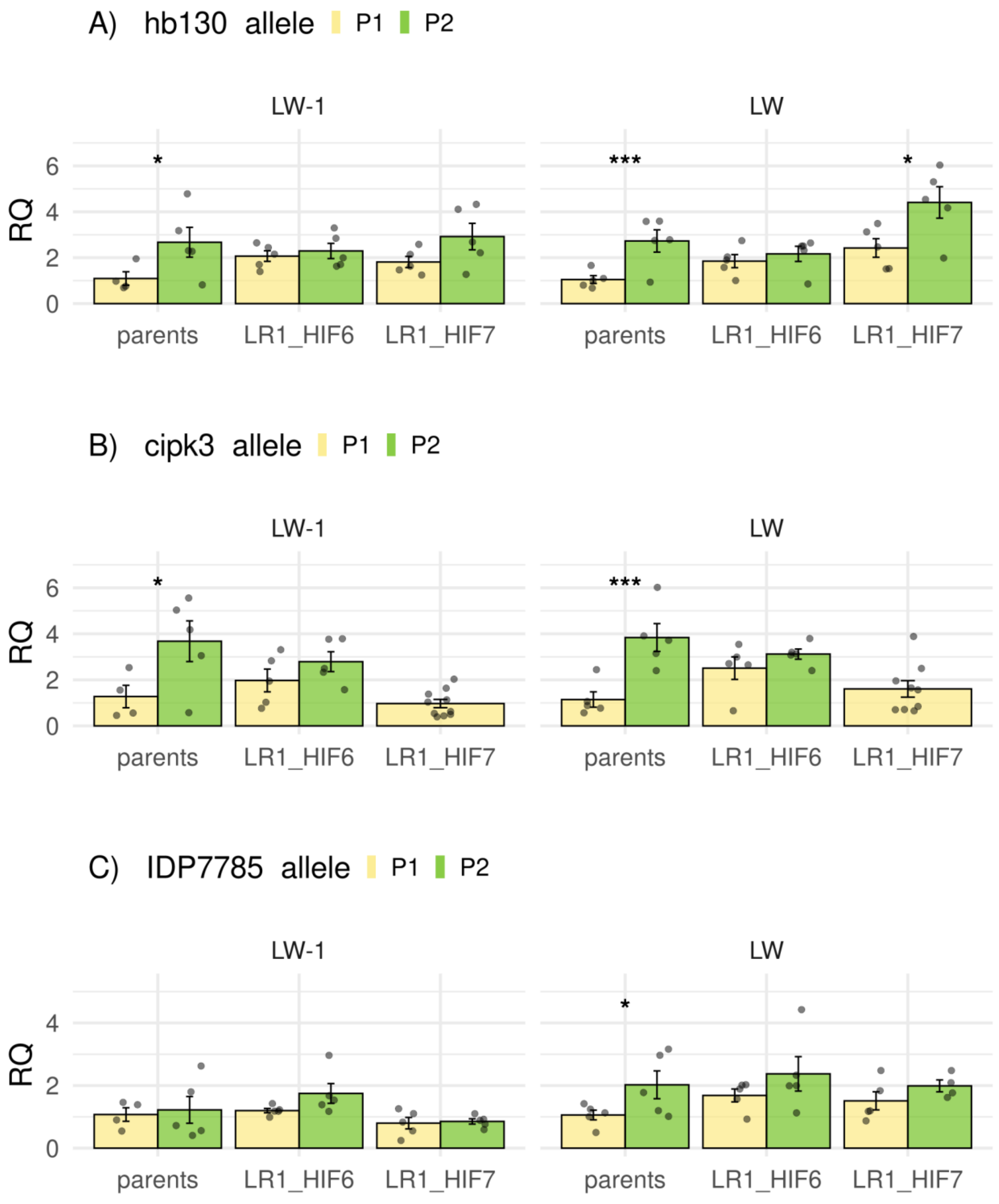

Supplementary Fig. S9. Transcript level measurements in candidate genes.
Relative quantification (RQ) of transcript abundance of three candidate genes *hb130* (**A**), *cipk3* (**B**), and *IDP7785* (**C**) in two root tissues from five biological replicates of P1, P2, HIF6_A, HIF6_B, HIF7_A and HIF7_B (see **Supplementary Fig. S4**). Tissues included the distal 10 cm of the excised roots of the previous last whorl (LW-1) and last whorl (LW). Individual dots indicate different biological replicates, bars show means ± SE and are coloured according to the allele of the respective gene. Significant differences between groups are indicated above the bars with stars: *** P < 0.01, ** P < 0.01, * P < 0.05.

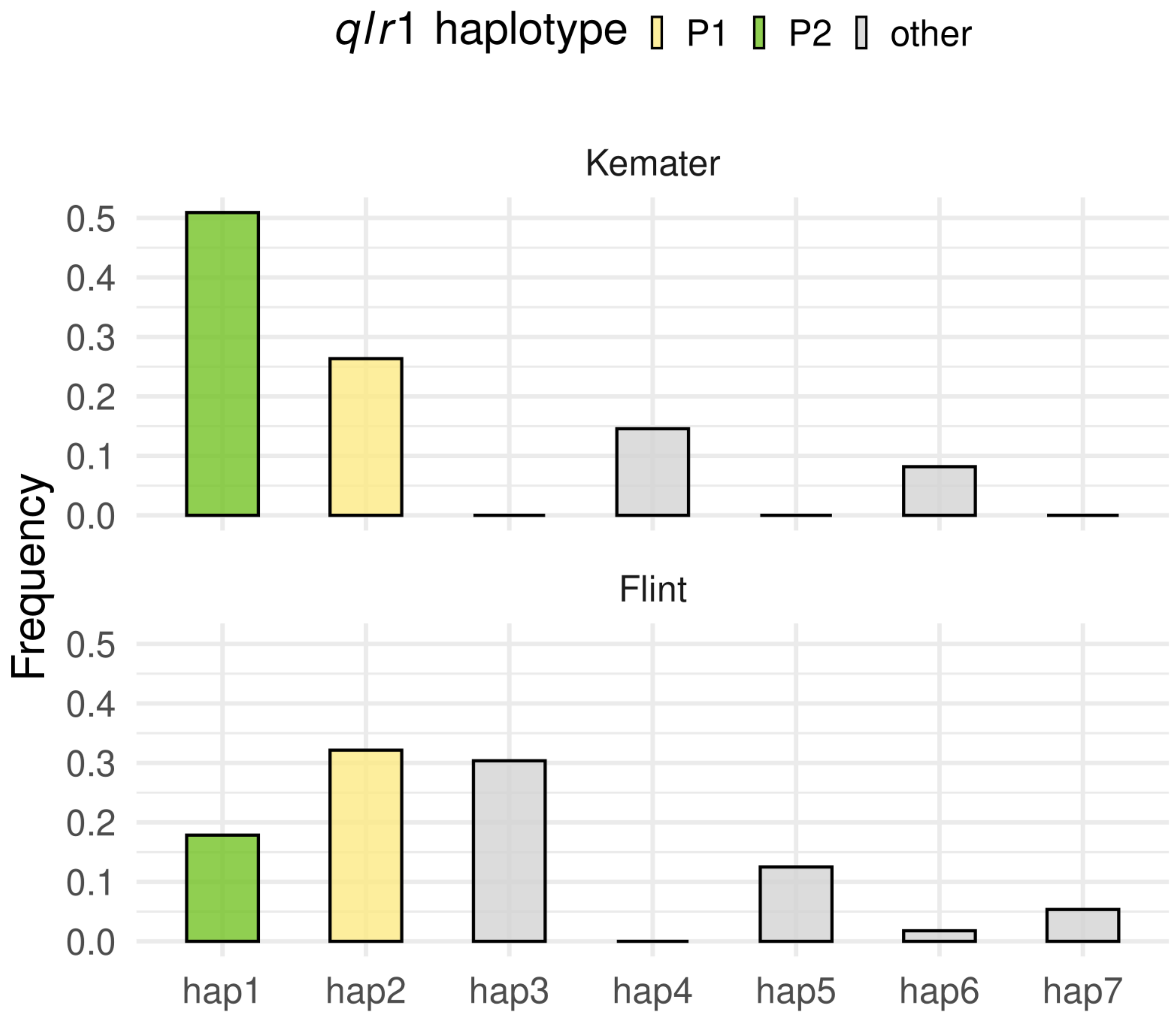

Supplementary Fig. S10. *qlr1* haplotype frequency in landraces and improved Flint lines. Bars show the haplotypes frequency at the *qlr1* locus in the Kemater landrace (n = 501) and improved Flint lines (n = 65). P1 and P2 haplotypes are coloured in yellow and green, respectively.
